# Cancer-educated mammary adipose tissue-derived stromal/stem cells in obesity and breast cancer: spatial regulation and function

**DOI:** 10.1186/s13046-022-02592-y

**Published:** 2023-01-30

**Authors:** Andreas Ritter, Nina-Naomi Kreis, Susanne Roth, Alexandra Friemel, Babek Kahn Safdar, Samira Catharina Hoock, Julia Maria Wildner, Roman Allert, Frank Louwen, Christine Solbach, Juping Yuan

**Affiliations:** Obstetrics and Prenatal Medicine, Gynecology and Obstetrics, University Hospital Frankfurt, J. W. Goethe-University, Theodor-Stern-Kai 7, D-60590 Frankfurt, Germany

**Keywords:** Breast adipose tissue-derived mesenchymal stromal/stem cells, Cancer-associated fibroblasts, Obesity, Breast cancer, Epithelial-to-mesenchymal transition, Chemoresistance, Cancer stem cells, Tumor microenvironment

## Abstract

**Background:**

Breast cancer is the most frequently diagnosed cancer and a common cause of cancer-related death in women. It is well recognized that obesity is associated with an enhanced risk of more aggressive breast cancer as well as reduced patient survival. Breast adipose tissue-derived mesenchymal stromal/stem cells (bASCs) are crucial components of the tumor microenvironment. A key step initially involved in this process might be the de-differentiation of bASCs into tumor supporting phenotypes.

**Methods:**

In the present work, we isolated bASCs from adipose tissues adjacent to the tumor (aT bASCs) from lean- (ln-aT bASCs, BMI ≤ 25) and breast cancer patients with obesity (ob-aT bASCs, BMI ≥ 35), and analyzed their phenotypes with functional assays and RNA sequencing, compared to their counterparts isolated from adipose tissues distant from the tumor (dT bASCs).

**Results:**

We show that ln-aT bASCs are susceptible to be transformed into an inflammatory cancer-associated phenotype, whereas ob-aT bASCs are prone to be cancer-educated into a myofibroblastic phenotype. Both ln-aT- and ob-aT bASCs compromise their physiological differentiation capacity, and upregulate metastasis-promoting factors. While ln-aT bASCs stimulate proliferation, motility and chemoresistance by inducing epithelial-mesenchymal transition of low malignant breast cancer cells, ob-aT bASCs trigger more efficiently a cancer stem cell phenotype in highly malignant breast cancer cells.

**Conclusion:**

Breast cancer-associated bASCs are able to foster malignancy of breast cancer cells by multiple mechanisms, especially, induction of epithelial-mesenchymal transition and activation of stemness-associated genes in breast cancer cells. Blocking the de-differentiation of bASCs in the tumor microenvironment could be a novel strategy to develop an effective intervention for breast cancer patients.

**Significance:**

This study provides mechanistic insights into how obesity affects the phenotype of bASCs in the TME. Moreover, it highlights the molecular changes inside breast cancer cells upon cell-cell interaction with cancer-educated bASCs.

**Graphical abstract:**

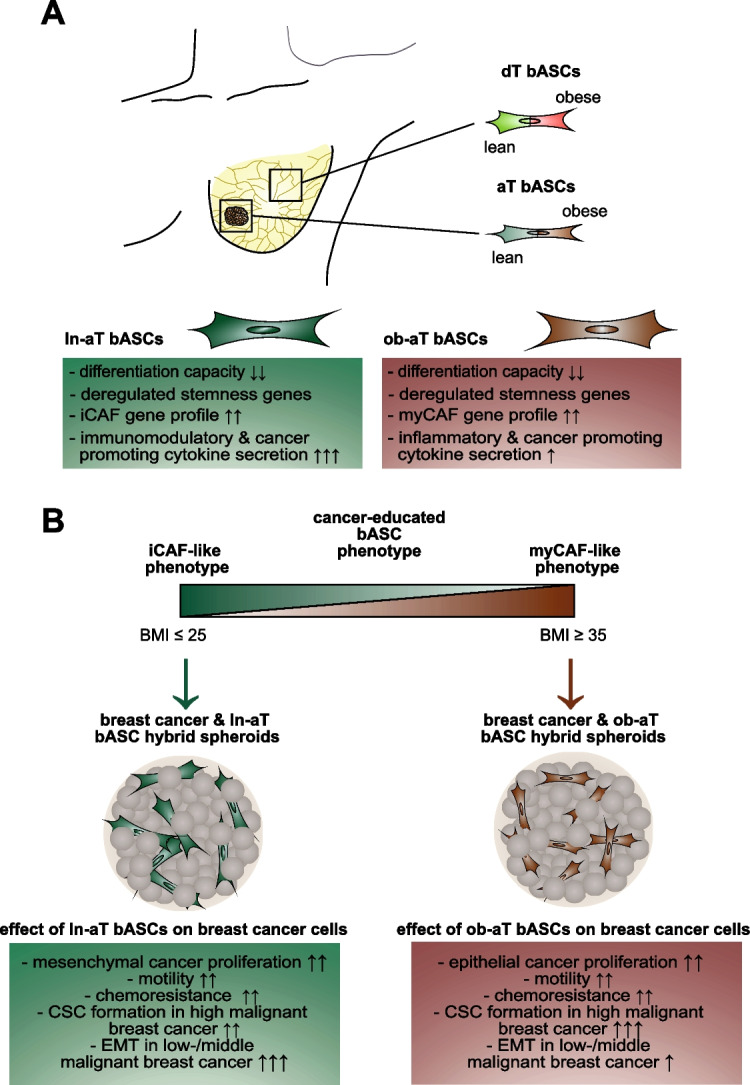

**Supplementary Information:**

The online version contains supplementary material available at 10.1186/s13046-022-02592-y.

## Background

The prevalence of obesity has been increasing in recent decades and has more than doubled worldwide since 1980 [1], posing a big challenge to the entire society and health care systems [2, 3]. Obesity is associated with enhanced risk of more aggressive breast cancer as well as reduced survival of postmenopausal breast cancer patients [4]. Despite intensive research, the relationship between obesity and breast cancer development is still not completely understood. Adipocyte hypertrophy during the development of obesity induces hypoxic conditions within the adipose tissue and results in chronic and systemic inflammation, endoplasmic reticulum (ER) stress, and altered extracellular matrix (ECM) composition [5]. In addition, obesity, indicated by body mass index (BMI) over 30, interferes with the immune system including the activation of macrophages via toll-like receptors 4 (TLR4), which stimulates nuclear factor kappa B (NFĸB) signaling und further fuels inflammation of adipose tissue [6]. These changes lead adipocytes and stromal cells to produce pro-inflammatory cytokines, including interleukin 6 (IL6), IL1β and tumor necrosis factor α (TNF-α), and growth factors, such as insulin, insulin-like growth factor 1 (IGF-1) and leptin [7, 8]. Increased cytokines and chemokines are further accompanied by increased infiltration of immune cells, cellular stress, hypoxia, insulin resistance, glucose intolerance, adipocyte hypertrophy and hyperplasia, and impaired tissue homeostasis [9]. Most of these enumerated alterations including increased growth factors, hypoxia and deregulated immune response are well known to promote breast cancer development and progression [10]. Although high serum levels of insulin, leptin and estradiol have been proposed to activate diverse signaling cascades and contribute to the progression of postmenopausal breast cancer of patients with obesity [4, 11], however, the molecular mechanisms by which obesity correlates with premenopausal breast cancer remain to be unraveled.

The tumor microenvironment (TME) is crucial for breast cancer progression, which has been the subject of intense research and is influenced by high grade of obesity [12]. Breast cancer is mainly surrounded by mammary adipose tissue, which contains a multitude of cell types, such as endothelial cells, progenitor cells, immune cells, fibroblasts and mesenchymal stromal/stem cells (MSCs), which, together with soluble factors as well as insoluble ECM proteins secreted by adipocytes, stromal cells and cancer cells, constitute the TME [13–15]. Among these cell types, fibroblasts have attracted high attention, as they are capable of de-differentiation into several types of cancer-associated fibroblasts (CAFs) in the TME of breast cancer, including a myofibroblastic (myCAF) and an inflammatory (iCAF) subgroup [16]. These CAFs promote tumor growth, invasion, metastasis and resistance to chemotherapeutics of breast cancer cells by secreting factors including CC-chemokine-ligand 2 (CCL2), C-X-C motif ligand 1–3 (CXCL1–3), and IL6, stiffening the ECM, affecting cancer cell metabolism [17], interacting with and influencing the functions of diverse stromal cells, such as MSCs, endothelial, and immune cells [16].

MSCs are a multipotent cell type and exist in diverse organs and tissues. They are involved in the maintenance of tissue homeostasis, regulation of local immune response, and repair of damaged tissues [18]. Beside their high differentiation capacity, they share many features with fibroblasts such as similar surface markers, morphology, gene expression profiles and proliferation rates [19]. As a result, much attention has also been paid to how different MSC subtypes contribute to breast cancer progression. However, most of these studies were based on MSCs isolated from non-breast sources, such as abdominal adipose tissue, bone marrow and peripheral blood [20–22]. MSCs from different tissues have distinct transcriptomic, biochemical, and secretory profiles as well as biological functions [23, 24]. This leads to bivalent results with tumor-supportive and -suppressive functions [25, 26], which may not reflect the in vivo situation in breast cancer tissue.

Little is known about the involvement of adipose tissue-derived mesenchymal stromal/stem cells (bASCs) isolated directly from mammary adipose tissues surrounding breast cancer. To investigate the bidirectional relationship between breast cancer cells and bASCs, we isolated bASCs from mammary adipose tissue adjacent (≤ 3 cm, −aT) to the tumors of lean (ln, BMI ≤ 25) or breast cancer patients with obesity (ob, BMI ≥ 35) and analyzed their phenotypes, compared to their control counterparts isolated from mammary adipose tissue distant to the tumors (≥ 9 cm, −dT) from the same patients. We show that these breast cancer associated bASCs highly reduce their differentiation capacity and alter their cytokine and chemokine secretion pattern. Interestingly, like fibroblasts, bASCs are also able to de-differentiate into at least two types of CAF-like cells that stimulate proliferation, motility and chemoresistance of low- as well as high-malignant breast cancer cells in 3D-spheroids, supporting the tumorigenic behavior of bASCs in the TME.

## Methods

### bASC isolation, cell lines, cell culture and differentiation

This study is approved by the Ethics Committee of the Johann Wolfgang Goethe-University Hospital Frankfurt (reference number: 443/11 and 4/09) and informed written consent was obtained from all donors. Mammary adipose tissue was obtained at least 9 cm distant from the tumor site (dT) and maximal 3 cm adjacent to the tumor site (aT) from the same women undergoing breast cancer surgery. Clinical information of patients is detailed in Table S1. bASCs were isolated as described [20] with modifications. In brief, obtained dT and aT adipose tissues were immediately washed, minced to small pieces, and digested with 1 mg/ml collagenase type I for 1 h, 200 rpm at 37 °C. Cells were pelleted by centrifugation at 700 g for 10 min and filtered through a 100 μm mesh filter to remove undigested adipose tissue. The remaining blood cells were lysed by addition of red blood cell lysis buffer (155 mM NH_4_Cl, 10 mM KHCO_3_, and 0.1 mM EDTA) for 10 min at 37 °C. To remove the lysis buffer, cells were centrifuged at 700 g for 10 min. The remaining cells were centrifuged, seeded onto 6-cm cell culture plates in DMEM containing 20% fetal bovine serum (FBS), 100 mg/ml streptomycin, 100 U/ml penicillin, 2 mM l-glutamine, and 1 g/ml amphotericin-B and cultured under standard cell culture conditions. After 24 h, non-adherent cells were removed and the remaining cells were washed, cultured, expanded and characterized (ln: Table S2, ob: Table S3). Early passages (P2 to P6) of isolated bASCs were used for experiments. The cells isolated from patients treated with tamoxifen (TMX) or radiotherapy were excluded for transcriptomic analysis. Breast cancer cell lines were chosen based on their cell surface receptor composition, namely estrogen (ER), progesterone (PR) and human epidermal growth factor receptor 2 (HER2) [27], and their malignancy potential (low/intermediate/high). The breast cancer cell lines BT474^(ER+, PR+, HER2+, low)^, MDA-MB-361^(ER+, PR+/−, HER2+, intermediate)^, MCF-7^(ER+, PR+, HER2+, low)^ and MDA-MB-231^(ER-, PR-, HER2-)^) were obtained from ATCC (Wesel, Germany). All cells were cultured as instructed.

The differentiation of bASCs toward adipogenic, osteogenic and chondrogenic lineages are detailed in supplementary information.

### Cell viability, cell cycle distribution and flow cytometry (FACS)

Cell viability, cell cycle distribution and FACS of bASCs and breast cancer cell lines are detailed in supplementary information.

### Indirect immunofluorescence staining, immunohistochemistry of breast cancer tissue, imaging and signal intensity measurement

The indirect immunofluorescence staining, immunohistochemistry staining of breast cancer tissue, image acquisition, primary and secondary antibodies and signal intensity quantification are detailed in supplementary information.

### Western blot analysis

Western blot analysis, primary and secondary antibodies are detailed in supplementary information.

### RNA extraction, real-time PCR (qPCR) and transcriptomic analysis (RNA-seq)

RNA extraction, qPCR, probes and RNA-seq are detailed in supplementary information**.**

### Human cytokine array and ELISA

Human cytokine array and ELISA quantifications are detailed in supplementary information**.**

### Spheroid formation, mitotic index,spheroid proliferation and epithelial-to-mesenchymal transition (EMT) analysis

The spheroid formation, mitotic index, EMT and spheroid proliferation assays are described in supplementary information and as reported [28].

### Motility assay

The motility assay is described in supplementary information and the patterns of motility were evaluated as reported [29].

### Spheroid cell viability, foci analysis and live/dead cell assay

Spheroid cell viability, foci analysis and live/dead cell assay are detailed in supplementary information**.**

### Statistical analysis

Before statistical analysis, an outlier test was performed with all data sets. Student’s t-test (two tailed and paired or homoscedastic) was used to evaluate the significance of difference between diverse groups for gene analysis, cell viability assay, cell cycle distribution and western blot analysis. The statistical evaluation of the single cell tracking assay, immunofluorescence quantification, spheroid evaluation, foci quantification, live/dead fluorescence quantification was performed by using an unpaired Mann-Whitney U test (two tailed). Difference is considered as statistically significant when *p* < 0.05.

## Results

### Lean and obese bASCs reduce their differentiation capacity within the TME

**Fig. 1:**
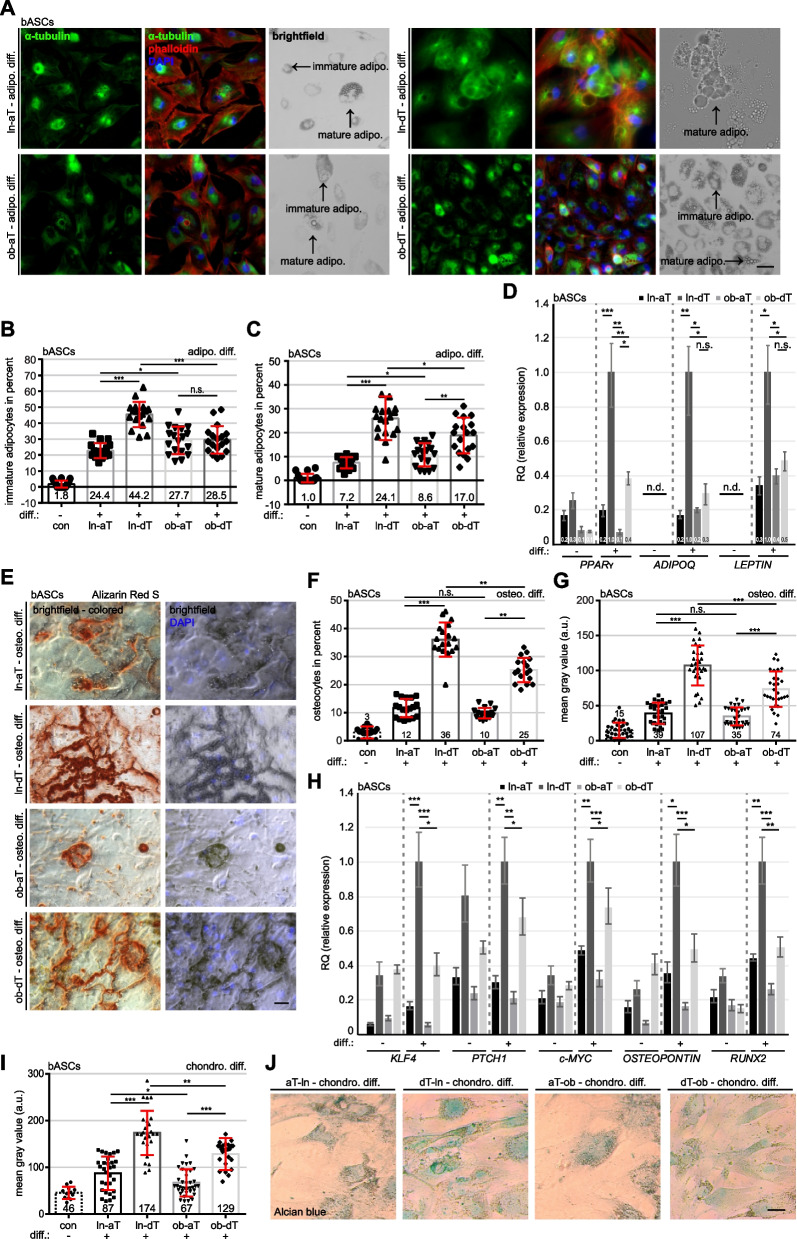
Lean and obese bASCs adjacent to breast cancer display an impaired differentiation capacity. **A-J** bASCs (ln-aT, ln-dT, ob-aT and ob-dT) were induced to adipogenic (adipo. Diff.) (**A**-**D**) for 14 days, osteogenic (osteo. Diff.) (**E**-**H**) and chondrogenic differentiation (chondro. Diff.) (**I** and **J**) for 21 days and their differentiation rates were evaluated. ln-dT bASCs were used as control cells without differentiation medium. **A** bASCs were stained for α-tubulin (green), phalloidin (red) to visualize the cytoskeleton, and DNA (DAPI, blue). Example images are shown. Scale bar: 50 μm. **B** and **C** The percentage of immature adipocytes (lipid vacuoles < 5 nm) (**B**) or mature adipocytes (lipid vacuoles > 5 nm) (**C**) was quantified. The results of individual bASC subgroups are presented as mean ± SEM (*n* = 500 cells for each condition, pooled from three experiments). **D** The gene expression of *PPARγ*, *ADIPOQ* and *LEPTIN* is shown for undifferentiated (−) and differentiated (+) bASCs. The results are from three individual experiments and presented as mean ± SEM. **E** bASCs were stained with Alizarin Red S to visualize calcium deposition. Representative images are shown. Scale bar: 50 μm. **F** and **G** The percentage of bASCs showing calcium deposition (**F**) and the mean gray value (**G**) were evaluated. The results are presented as mean ± SEM (F: n = 500 cells for each condition, pooled from three experiments, G: *n* = 30 images for each condition, pooled from three experiments). **H** The gene expression of *KLF4*, *PTCH1*, *c-MYC*, *OSTEOPONTIN* and *RUNX2* is shown for undifferentiated (−) and differentiated (+) bASCs. The results are from three individual experiments and presented as mean ± SEM. **I** and **J** bASCs were stained with Alcian blue to visualize acidic polysaccharides. Representative bright-field images are shown (**J**). Scale bar: 50 μm. The quantification of the mean gray value is presented (**I**). The results are shown as mean ± SEM (*n* = 30 images for each condition, pooled from three experiments). Unpaired Mann-Whitney U test was used in (**B** and **C**), (**F** and **G**) and (**I**). ∗*p* < 0.05, ∗∗*p* < 0.01, ∗∗∗*p* < 0.001. Student’s t test was used in (**D**) and (**H**). ∗*p* < 0.05, ∗∗*p* < 0.01, ∗∗∗*p* < 0.001

The importance of the differentiation capacity of MSCs is well-known for proper tissue homeostasis and regeneration [30, 31]. We questioned how the TME would affect the differentiation capacity of ln-aT bASCs and ob-aT bASCs, compared to their counterpart ln-dT bASCs and ob-dT bASCs isolated from adipose tissue distant to the tumors. Clinical information of the patients is listed in Table S1. These bASCs were subjected to adipogenic, osteogenic and chondrogenic differentiation stimuli and their differentiation capacities were evaluated by lineage specific staining and gene analysis. The analysis showed that ob-bASCs had a highly reduced differentiation capacity in all three analyzed lineages compared to ln-bASCs (Fig. 1), supporting our previous reports of visceral and subcutaneous ASCs [9, 32, 33]. Specifically, both ln- and ob-aT bASCs displayed a significantly reduced adipogenic differentiation capacity compared to ln- and ob-dT bASCs (Fig. 1A-D). In accordance, ln-dT bASCs had the highest percentage of immature- (44.2%) and mature adipocytes (24.1%), compared to the other three subgroups (Fig. 1A-C). Although ob-dT bASCs showed no difference in the percentage of immature adipocytes relative to ob-aT bASCs (ob-dT bASCs: 28.5% vs. ob-aT bASCs: 27.7%) (Fig. 1A and B, 4^th^ and 5^th^ bars), the ob-dT bASCs displayed a significantly increased number of mature adipocytes (ob-dT bASCs: 17.0% vs. ob-aT bASCs: 8.6%) (Fig. 1A and C, 4^th^ and 5^th^ bars), suggesting a reduction of adipogenic differentiation ability of ob-aT bASCs. In line with this observation, the expression of adipogenic-related genes such as peroxisome proliferator-activated receptor γ (*PPARγ)*, adiponectin (ADIPOQ) and *LEPTIN* were highly upregulated by 66–83% in ln-dT bASCs compared to ln-aT bASCs (Fig. 1D, 5^th^ vs. 6^th^ bar). Reduced adipogenic gene expression was also observed in ob-aT bASCs in comparison to ob-dT bASCs (Fig. 1D, 7^th^ vs. 8^th^ bar), yet not to the same extent as in ln bASCs, and a significant difference was observed only for *PPARγ*.

Similar results were obtained from microscopic analyses of the calcium deposition in differentiated osteocytes stained with Alizarin Red S (Fig. 1E). Compared to dT bASCs, aT bASCs demonstrated a reduced percentage of differentiated osteocytes and a lower mean gray value, a parameter used for the evaluation of the staining signal [34], of the Alizarin Red S staining (Fig. 1E-G, 2^nd^ vs. 3^rd^ bar and 4^th^ vs. 5^th^ bar). In particular, ob-dT bASCs had a decreased differentiation capacity by 11% (Fig. 1E-G, 3^nd^ vs. 5^th^ bar). Consistent with these results, all five genes *KLF4*, *PTCH1*, *c-MYC*, *OSTEOPONTIN* and *RUNX2* associated with osteogenic differentiation were significantly higher in ln- and ob-dT bASCs compared to ln- and ob-aT bASCs (Fig. 1H). In further support, the chondrogenic differentiation, indicated with the specific staining of Alcian blue used to identify sulfated proteoglycans, showed a comparable trend (Fig. 1I and J). bASCs in the proximity of breast cancers exhibited drastically reduced chondrogenic differentiation capacity, as indicated by a weaker staining of polysaccharides quantified by the mean gray value (Fig. 1I, 2^nd^ and 4^th^ bars). Both distant bASCs subgroups displayed a moderate blue staining, whereas ln-dT bASCs showed a 26% increased gray value compared to ob-dT bASCs (Fig. 1I, 3^rd^ vs. 5^th^ bars). These results clearly demonstrate an impaired differentiation capability of obese- and cancer-near bASCs, suggesting a cancer-educated phenotype of bASCs in the TME of breast cancers.

To exclude the possibility that these differentiation alterations were a result of a changed cell cycle distribution or cell proliferation, cell cycle analyses and cell viability assays were performed. None of the analyzed bASC groups (ln/ob-aT/dT) showed significant differences in both assays (Fig. S1A and B). Moreover, all bASCs subgroups displayed a comparable CD (cluster of differentiation) marker profile specific for MSCs (Table S2 and S3) [35].

### bASCs de-differentiate into distinct CAF-like phenotypes

**Fig. 2:**
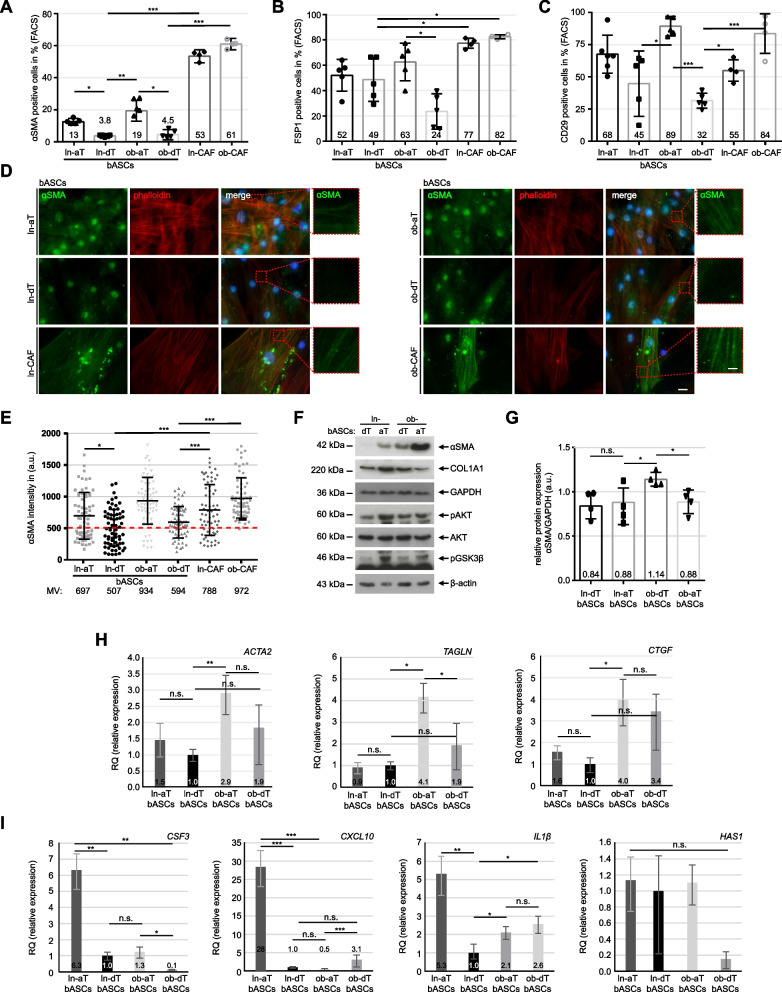
The tumor microenvironment induces the de-differentiation of bASCs. **A-C** bASCs (ln-aT, ln-dT, ob-aT and ob-dT) were stained for αSMA, FSP1 and CD29 for FACS analyses. CAFs (ln-CAF and ob-CAF) were isolated and stained as positive controls. Quantification of αSMA (**A**), FPS1 (**B**) and CD29 (**C**) are shown as bar graphs. The results are from three independent experiments (*n* = 3, 60.000 cells for each condition and in each group) and presented as mean ± SEM. **D** Representative images of bASCs and CAFs stained for αSMA (green), phalloidin (red) and DNA (DAPI, blue) are shown. Red boxes indicate measured areas. Scale: 25 μm. Inset scale: 12.5 μm. **E** The evaluation of the mean fluorescence intensity of αSMA is presented as scatter plots. The results are from three independent experiments (*n* = 3, 90 cells for each condition and in each group) and presented as mean ± SEM. **F** Cellular extracts from bASCs were prepared for WB analysis with antibodies against αSMA, COL1A1, caveolin-1, AKT, pAKT and pGSK3β. GAPDH and β-actin served as loading controls. **G** Quantification of the αSMA signal in WB is shown, relative to the corresponding amount of GAPDH. The results are from three independent experiments and presented as mean ± SEM. **H** and **I** Relative gene levels of *ACTA2*, *TAGLN* and *CTGF,* important myCAF marker genes (**H**), and relative gene expression of *CSF3*, *CXCL10*, *IL1β* and HAS1, iCAF marker genes (**I**), are shown for bASC subgroups (ln-dT, ln-aT, ob-dT and ob-aT). The results are from three independent experiments and presented as bar graphs with mean ± SEM. Student’s t test was used in (**A**-**C**) and (**G**-**I**). Unpaired Mann-Whitney U test was used in (**E**). ∗*p* < 0.05, ∗∗*p* < 0.01, ∗∗∗*p* < 0.001. ∗*p* < 0.05, ∗∗∗*p* < 0.001

Tumors are known to shape their TME to support their development, proliferation, metastasis and therapeutic resistance [36]. Especially, CAFs derived from various cell origins such as fibroblasts and MSCs [16, 37] are key components of the TME and important in TME remodeling [38]. To address if aT-bASCs de-differentiate into a myofibroblastic cancer-associated (myCAF) phenotype, we assessed their specific protein markers alpha-smooth muscle actin (αSMA), fibroblast-specific protein1 (FSP1) and integrin subunit beta 1 (CD29) by flow cytometry. As presented in Fig. 2A-C, ln-aT bASCs showed a moderately increased expression of αSMA and CD29 compared to ln-dT bASCs (Fig. 2A and C, 1^st^ vs. 2^nd^ bar). FSP1 did not show any differences in these lean bASCs subtypes (Fig. 2B, 1^st^ vs. 2^nd^ bar). Interestingly, ob-aT bASCs displayed a significantly enhanced expression of all three myCAF marker proteins in comparison to ob-dT bASCs (Fig. 2A-C, 3^rd^ vs. 4^th^ bar). CAFs, isolated from the same breast cancer as reported [39] and served as positive control, showed a remarkably high expression of αSMA and FSP1 (Fig. 2A and B, 5^th^ and 6^th^ bar), whereas CD29 was only upregulated in CAFs isolated from patients with obesity, similar to ob-aT bASCs (Fig. 2C, 3^th^ and 6^th^ bars).

To corroborate these results, bASCs were stained for αSMA, vimentin (VIM) and collagen type I alpha 1 chain (COL1A1) for intensity quantification. The analyses revealed elevated signals for αSMA, VIM and COL1A1 in ln/ob-aT bASCs (Fig. 2D and E, Fig. S1C-F), whereas ln/ob-dT bASCs only displayed low to moderate staining signals (Fig. 2D and E, Fig. S1C-F). Of note, the expression of COL1A1 in bASCs could be clustered into three groups (Fig. S1C, E and F): the majority of lean and obese dT bASCs had a low expression of COL1A1 (Fig. S1C and F, 1st and 3rd bars), a high expression was observed in ln/ob-aT bASCs (Fig. S1C and F), and a high percentage of ob-aT bASCs also presented intermediate levels (Fig. S1C and F).

Furthermore, we analyzed the protein levels of αSMA, COL1A1, pAKT, AKT and pGSK3β via Western blot analysis. ob-aT bASCs presented an upregulated αSMA compared to all other bASC subgroups (Fig. 2F and G). By contrast, αSMA was just marginally increased in ln-aT bASCs (Fig. 2F and G), but COL1A1 was increased in ln-aT bASCs as indicated by the previous immunofluorescence staining (Fig. 2F and Fig. S1C, E and F). Moreover, ln-aT bASCs, like ob-aT bASCs, showed an activated PI3K pathway evidenced by increased pAKT and pGSK3β (Fig. 2F).

Unlike ob-aT bASCS, ln-aT bASCs displayed an inconclusive expression of specific myCAF markers. We hypothesized that ob-aT bASCs represented a myCAF-like type, whereas ln-aT bASCs might exhibit a different cancer-associated phenotype, namely iCAF, as described for fibroblasts [40, 41]. To clarify this issue, gene expression analysis of the myCAF associated genes *ACTA2*, connective tissue growth factor (*CTGF)* and transgelin (*TAGLN),* and iCAF classified genes colony stimulating factor 3 (*CSF3)*, *CXCL10*, *IL1β* and hyaluronan synthase 1 (*HAS1)* [40, 42] were performed. Indeed, these analyses revealed that ob-aT bASCs had a predominant myCAF phenotype with highly increased gene levels of *ACTA2*, *TAGLN* and *CTGF* compared to ln-dT bASCs (Fig. 2H, 2^nd^ vs. 3^rd^ bar). Strikingly, though displaying slightly elevated gene profiles of *ACTA2* and *CTGF* (Fig. 2H, 1^st^ vs. 2^nd^ bar), ln-aT bASCs had a significantly increased expression of iCAF genes including *CSF3*, *CXCL10* and *IL1β* (Fig. 2I, 1^st^ vs. 2^nd^ bar). The gene levels of *HAS1* were low and not significantly altered in bASCs, because of the low gene copy number (Fig. 2I, 4^th^ graph). Ob-aT bASCs mostly had lower expression of these genes compared to other bASCs (Fig. 2I). These results suggest that bASCs in the TME are de-differentiated toward CAF-like phenotypes, which explains the greatly reduced differentiation capacity of both bASCs subgroups. Furthermore, these data highlight that obesity impacts the de-differentiation of bASCs isolated from adipose tissue near the breast tumor. Of importance, ln-aT bASCs reflected the gene expression pattern of an iCAF-like phenotype, whereas ob-aT bASCs demonstrated a myCAF gene and protein profile.

### Transcriptional reprogramming of bASCs in the TME

**Fig. 3:**
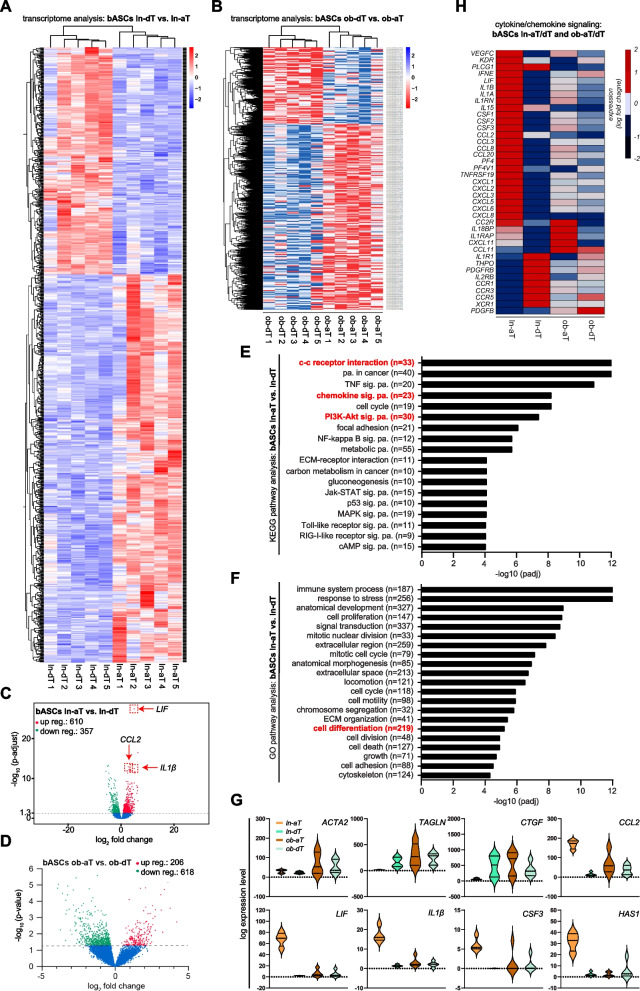
Transcriptomic profiles of lean and obese bASCs adjacent to breast cancers. **A**-**G** Total RNAs were extracted from each sample of bASC subgroups (ln-dT, ln-aT, ob-dT and ob-aT, 5 samples for each subgroup) for transcriptome analysis. **A** and **B** Heatmap of significantly differentially expressed genes of ln-dT vs. ln-aT bASCs (**A**) and ob-dT vs. ob-aT (**B**). Gene expression was analyzed using DESeq2 R package. Genes with a *p*-value < 0.01, and a fold change greater than 2 (red color code) and below − 2 (blue color code), respectively, were included. **C** and **D** Volcano plots showing the adjusted *p*-value (adj. *p* > 0.05) for genes differentially expressed between bASCs close to and distant to the breast cancers in lean (**C**) and obese (*p* > 0.05) (**D**) bASCs. Upregulated genes are depicted in red color, downregulated in green, and non-changed genes in blue (adjusted *p*-value > 0.05). **E** and **F** Significantly enriched KEGG pathways (**E**) and GO pathways (**F**) are presented for ln-aT bASCs compared to ln-dT bASCs. For each KEGG or GO pathway, the bar shows the adjusted *p*-value. The numbers (n) behind the pathway names indicate deregulated genes. **G** Violin plots present selected myCAF/iCAF genes differentially expressed. Values reflect the log expression levels of genes from the RNA-seq data. **H** Heatmap depicts significantly differentially expressed cytokine/chemokine genes in four bASCs subgroups. These genes have a fold change greater than 2 (red color code) or below − 2 (blue color code). Genes in (**A**-**F**) have an adjusted *p*-value of ≤0.05 and genes shown in (**G**) have an adjusted *p*-value of ≤0.05, at least for one condition (ln-aT or ob-aT)

Since ln/ob-aT bASCs displayed a functional decline and a transition to cancer-educated bASC phenotypes, a transcriptomic analysis should reveal the impact of the TME on the overall gene expression of aT bASCs in comparison to dT bASCs. Total RNAs were extracted from the four bASC subgroups for whole-genome mRNA-sequencing (RNA-seq) [43]. Strikingly, the transcriptome analysis revealed 967 deregulated genes with a significantly adjusted *p*-value in ln-aT bASCs versus ln-dT bASCs visualized by a heatmap (Fig. 3A) and a volcano plot (Fig. 3C). The transcriptome comparison between ob-aT bASCs and ob-dT bASCs revealed 824 significantly deregulated genes by using the *p*-value without adjustment (Fig. 3B and Fig. 3D), possibly due to distinct gene alterations in each obese individual. The “Kyoto Encyclopedia of Genes and Genomes” (KEGG) pathway analysis highlighted the most significant changes in their gene expression of ln-aT bASCs in the cell-cell receptor interaction (33 genes), pathways in cancer (40 genes), TNF signaling pathway (20 genes), PI3K-Akt signaling pathway (30 genes), and the chemokine signaling pathway (23 genes), compared to ln-dT bASCs (Fig. 3E). Interestingly, a further pathway analysis by the “Gene Ontology project” (GO) revealed that pathways involved in immune system processes (187 genes), response to stress (256 genes), signal transduction (337 genes) and cell differentiation (219 genes) were altered in ln-aT bASCs (Fig. 3F). The altered genes in the cell differentiation pathway included stemness- and proliferation-associated genes such as Erb-B2 receptor tyrosine kinase 4 (*ERBB4)*, roundabout guidance receptor 2 (*ROBO2)*, slit guidance ligand 2 (*SLIT2)*, forkhead box C2 (*FOXC2*), SRY-box transcription factor 9 (*SOX9)*, hyaluronan synthase 2 (*HAS2)*, retinoblastoma-associated protein 1 (*E2F1)*, forkhead box L1 (*FOXL1)* and nuclear factor kappa b subunit 2 (*NFĸB2)*, all of which were upregulated in ln-aT bASCs (Fig. S2A, 1^st^ to 9^th^ plots). Their downregulated genes included platelet derived growth factor receptor beta (*PDGFRβ)*, androgen receptor (*AR)* and homeobox A2 (*HOXA2)* (Fig. S2A, 10^th^ to 12^th^ plots).

Transcriptome analysis corroborated the gene expression of myCAFs (Fig. 3G, 1^st^ to 3^rd^ plots). Important myCAF genes, including *ACTA2*, *TAGLN* and *CTGF,* were upregulated in ob-aT bASCs (Fig. 3G, 1^st^ to 3^rd^ plots), whereas ln-aT bASCs showed an increased expression of iCAF associated genes such as *CCL2*, leukemia inhibitory factor (*LIF)*, *IL1β*, *CSF3* and *HAS1* (Fig. 3G, 4^th^ to 8^th^ plots). In addition, ln-aT bASCs displayed various upregulated cytokine genes including *IL8*, *CXCL1–3* and *CXCL10* reported for iCAFs (Fig. S2B). Moreover, presented with a heatmap, ln-aT bASCs displayed a prominent iCAF phenotype by showing an upregulated gene expression of chemokines, including *CCL*, colony stimulating factor (*CSF*), C-C motif chemokine receptor 5 (*CCR5*), *CXCL*, cytokines such as *IL1A*, *IL1B*, *IL18BP*, the IL1 receptor accessory protein (*IL1RAP*), *LIF*, and growth factors including vascular endothelial growth factor C (*VEGFC*), *PDGFB* and its receptor *PDGFRB* (Fig. 3H). In sum, the transcriptome analysis supports the notion that the TME greatly impacts the function and biology of bASCs. In particular, the TME educates ln-aT bASCs into an iCAF-like phenotype displaying a high gene expression of cytokines, chemokines and growth factors.

### dT bASCs alter their cytokine secretion upon co-culture with triple negative breast cancer cells

Transcriptome analysis revealed a network of deregulated cytokines in bASCs isolated adjacent to breast cancer cells (Fig. 3H). To look at the secretion profiles of these cytokines, the conditioned media from bASCs were collected for evaluation using a human cytokine array containing 120 different targets. ln-aT as well as ob-aT bASCs secreted a higher amount of important migration and invasion stimulatory cytokines such as CXCL5, CXCL6, CXCL11, CCL27, IL8, the tissue inhibitor of metalloproteinase 1 (TIMP-1), the macrophage migration inhibiting factor (MIF), and the interleukin 6 signal transducer (IL6ST, also known as gp130 and IL6R-β) (Fig. S3A and B). CXCL11 was the only cytokine, which was significantly upregulated in ob-aT bASCs compared to ob-dT bASCs (Fig. S3B, 2^nd^graph).

To investigate the impact of breast cancer cells on the cytokine secretion of bASCs, all subgroups of bASCs were indirectly co-cultured (Fig. S2C) with the triple negative breast cancer cell line MDA-MB-231 for 7 days. The cells were then cultured in serum-free medium for additional 3 days for collecting conditioned media for cytokine evaluation. Surprisingly, 7-day indirect co-culture with MDA-MB-231 cells was sufficient to induce an altered secretion pattern in dT bASC control cells (Fig. S3A, 2^nd^ vs. 6^th^, and 4^th^ vs. 8^th^ row). By contrast, this indirect co-culture did not influence aT bASCs to the same extent as dT ASCs (Fig. S3A, 1^st^ vs. 5^th^ and 3^rd^ vs. 7^th^ row). Intriguingly, both ln/ob dT-bASCs significantly increased their secretion of stem cell factor (SCF), CCL5, VEGFA, oncostatin M (OSM), MIF, and osteoprotegerin (OPG) (Fig. S3C). In addition, the secretion of CXCL1 and CXCL5, two cytokines involved in breast cancer metastasis [44, 45], was moderately increased in all subgroups after indirect co-culture with MDA-MB-231 cells (Fig. S3D). These results highlight the importance of the interaction between breast cancer cells, the TME and bASCs, even an indirect short co-culture in this experimental setup, already partially educate dT bASCs by altering their secretion pattern of various cytokines.

### Ln- and Ob-aT bASCs upregulate the gene expression and secretion of tumor promoting cytokines

**Fig. 4:**
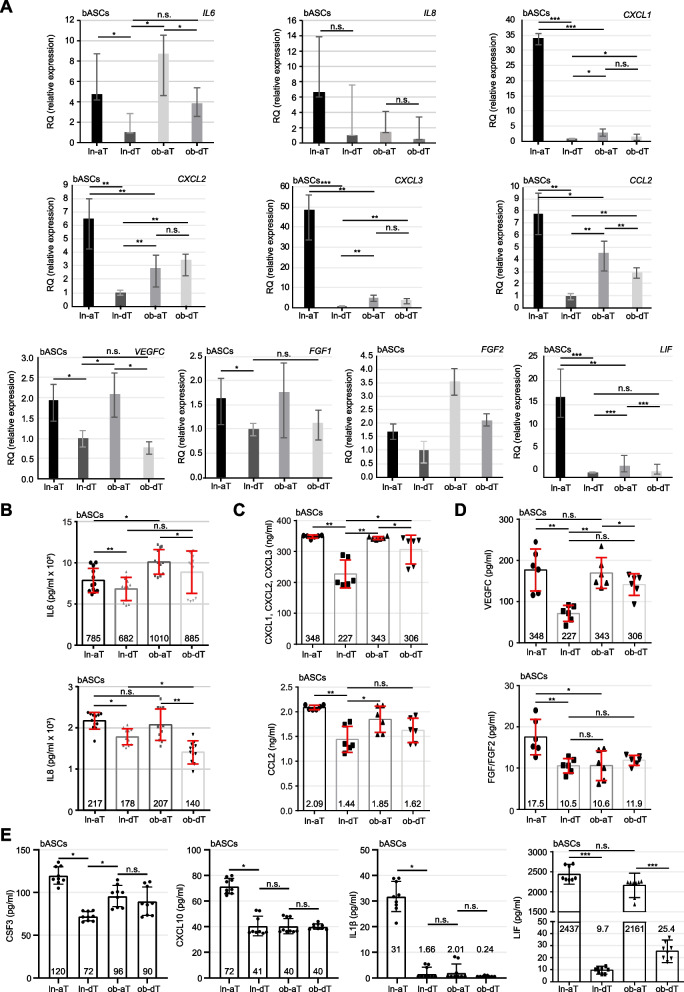
bASCs adjacent to breast cancer cells have increased gene expression and protein secretion of cancer promoting cytokines. **A** Relative gene levels of *IL6*, *IL8*, *CXCL1*, *CXCL2*, *CXCL3*, *CCL2*, *VEGFC*, *FGF1*, *FGF2*, and LIF are shown for bASC subgroups (ln-dT, ln-aT, ob-dT and ob-aT). The results are from three independent experiments and presented as bar graphs with mean ± SEM. **B**-**E** ELISA assays were performed with conditioned media from bASCs subgroups and the levels of cytokines IL6 and IL8 (**B**), chemokines CXCL1–3 and CCL2 (**C**), growth factors VEGFC and FGF1/2 (**D**), and inflammatory cancer-associated cytokines CSF3, CXCL10, IL1β and LIF (**E**) were analyzed. The results are from three independent experiments and presented as scatter bar graphs with mean ± SEM. Student’s t test was used. ∗*p* < 0.05, ∗∗*p* < 0.01, ∗∗∗*p* < 0.001

To further corroborate the transcriptome analysis data, we extracted RNA from four bASCs subgroups for quantitative gene analysis. Compared to ln-dT bASCs, ln-aT bASCs showed increased gene expression of *IL6*, *IL8*, *CXCL1–3*, *CCL2*, *VEGFC*, fibroblast growth factor 1 (*FGF1*) and *FGF*2, and *LIF* (Fig. 4A, 1^st^ vs. 2^nd^ bar) supporting the finding from the transcriptome analysis (Fig. 3H and Fig. S2B). The most significant hits were *LIF*, *CXCL1, CXCL2* and *CXCL3*, which were all highly upregulated with RQ values of 6.5 to 48.4 in ln-aT bASCs compared to the ln-dT bASCs (Fig. 4A). Remarkably, ob-aT bASCs showed a similar trend with an increased gene expression of *LIF* (RQ: 2.4), *CXCL1* (RQ: 3.0), *CXCL3* (RQ: 2.8), *CCL2* (RQ: 4.5), *FGF1* (RQ: 1.8), FGF2 (RQ: 3.5) and *VEGFC* (RQ: 2.1), but not to the extent of ln-aT bASCs (Fig. 4A). Strikingly, ob-dT bASCs already displayed an enhanced gene expression of *CXCL2*, *CXCL1*, *CXCL3*, *FGF2* and *CCL2,* compared to ln-dT bASCs (Fig. 4A), suggesting a crucial impact of obesity on bASCs.

To examine cytokine secretion, ELISA assays were performed with conditioned media from individual bASC subgroups. IL6 was upregulated in ob bASCs, whereas IL8 was slightly downregulated (Fig. 4B). Both ln-aT bASCs and ob-aT bASCs secreted high levels of cytokines (Fig. 4B). Additionally, ln-aT bASCs, compared to ln-dT bASCs, displayed significantly increased levels of CCL2, CXCL1–3, FGF1, FGF2 and VEGFC (Fig. 4C and D, 1^st^ vs. 2nd bars). As observed by gene analysis (Fig. 4A), ob-aT bASCs also secreted increased levels of CCL2, CXCL1–3 and VEGFC, in comparison to ob-dT bASCs (Fig. 4C and D, 3^rd^ vs. 4th bars), but not to the same extent as ln-aT bASCs did (Fig. 4C and D, 1^st^ vs. 3^rd^ bar).

To corroborate the gene expression data from transcriptomic analysis that suggest an iCAF phenotype for ln-aT bASCs, we analyzed the secretion of inflammatory cytokines and chemokines. Indeed, ln-aT bASCs had significantly increased secretion of CSF3, CXCL10 and IL1β, compared with all three other subgroups (Fig. 4E, 1^st^ to 3^rd^ graphs). Of importance, ob-dT bASCs were highly capable of releasing LIF with a value of 25.4 pg/ml relative to ln-dT bASCs with values of 9.7 pg/ml (Fig. 4E, 4^th^ graph, 2^nd^ and 4^th^ bars), whereas ln- and ob-aT bASCs secreted it in a significantly high amount with 2.4 ng/ml in ln-aT bASCS and 2.2 ng/ml in ob-aT bASCs (Fig. 4E, 4^th^ graph, 1^st^ and 3^rd^ bars). These data underscore the findings from the transcriptome analysis that ln-aT bASCs are de-differentiated into an iCAF-like phenotype. Importantly, most of these cytokines are tumor and metastasis promoting [16, 46, 47], suggesting that one of the main molecular mechanisms how ln/ob-aT bASCs support tumor development is via paracrine signaling.

### Ob- and ln-aT bASCs promote the growth of breast cancer spheroids via cell-cell contact

To study the effect of bASCs and their secreted cytokines, spheroids were generated with two luminal B breast cancer cell lines BT474^(ER+, PR+, HER2+)^ and MDA-MB-361^(ER+, PR+/−, HER2+)^ [27] and seeded on a bASC feeder layer as indicated in Fig. S3E. Area and diameter of spheroids were measured up to 96 h. The spheroids were then fixed, stained for phospho-histone H3 (pHH3_,_ Ser10), a mitotic marker, α-tubulin and DNA (DAPI), and the mitotic index and cell number were quantified. Low malignant BT474 spheroids, which were grown in a direct co-culture setup on a feeder layer of ln-aT bASCs, showed a growth advantage at 24, 48, 72 and 96 h, compared to a feeder layer formed by ln-dT bASCs (Fig. S4A). The area and diameter were significantly increased in BT474 spheroids, especially at early time points 24 and 48 h (Fig. S4A, C and E). Interestingly, BT474 spheroids co-cultured with ob-aT bASCs displayed an even greater growth advantage compared to ln/ob-dT bASCs (Fig. S4A-F). The direct comparison of lean and obese aT bASCs showed that ob-aT bASCs led to an increased growth rate of BT474 spheroids by 29.97% at 72 h and 27.83% at 96 h compared to ln-aT bASCs (Fig. S5A, black scatter plots), whereas there was no significant difference between lean and obese dT bASCs (Fig. S5A, gray scatter plots). The similar effect was observed for BT474 spheroid diameter, which increased by 18 to 21% over the 72- to 96 h period after co-culture with ob-aT bASCs, compared to ln-aT bASCs (Fig. S5B, black scatter plots). In addition, the diameter of BT474 organoids co-cultured with ob-dT bASCs increased significantly by 13%, compared with ln-dT bASCs (Fig. S5B, gray scatter plots). In line with this, spheroids co-cultured with ob-aT bASCs had a higher cell number and mitotic index than with ln-aT bASCs (Fig. S4G-J, black bars). Only moderate differences were observed on breast cancer cell spheroids co-cultured with lean or obese dT bASCs (Fig. S4G-J, gray bars).

An increased diameter was also observed in MDA-MB-361 spheroids directly co-cultured with aT bASCs (Fig. S5C-J). The growth benefit of spheroids has been further significantly enhanced by co-culture with ob-aT bASCs, compared to ln-aT bASCs (Fig. S5G and H). Like BT474 spheroids, the mitotic index and cell number were also increased in MDA-MB-361 spheroids co-cultured with ob-aT bASCs (Fig. S6A-D, black bars). A moderate promoting effect was observed in the spheroids co-cultured with lean and obese dT bASCs (Fig. S5C-J and S6A-D). These data support previous reports that visceral ASCs had a stimulatory effect on cancer cell proliferation in a direct co-culture setup [20, 48]. Collectively, aT bASCs have a highly increased capacity to fuel the growth of low to intermediate malignant breast cancer spheroids, which is further exacerbated by the obese state.

### The supernatant of aT bASCs stimulates breast cancer cell motility

A number of chemokines such as CXCL1–3, CXCL10 and CCL2 released from aT-bASCs are known to promote invasion and metastasis [49]. To examine this issue, epithelial MCF7^(ER+, PR+, HER2+)^ and mesenchymal MDA-MB-231^(ER-, PR-, HER2-)^ breast cancer cells incubated with supernatants of bASCs were tracked using time-lapse microscopy. The accumulated distance and the velocity of single-tracked cells are commonly used to assess the migratory capacity of cancer cells [50]. Supernatants of both ln- and ob-aT bASCs significantly increased the accumulated distance (ln-aT bASCs: 606 μm; ob-aT bASCs: 615 μm) and the velocity (ln-aT bASCs: 0.90 μm/s; ob-aT bASCs: 0.92 μm/s) of MCF7 cells, compared with control MCF7 cells, which had an accumulated distance of 363 μm and a velocity of 0.54 μm/s (Fig. S6E-G, 1^st^, 2^nd^ and 4^th^ scatter plots). Consistent with the proliferation results (Fig. S4 and S5), the supernatants of ln/ob-dT bASCs displayed moderate effects on MCF-7 cells with increased accumulated distances of 490 μm and 556 μm (Fig. S6F, 3^rd^ and 5^th^ scatter plots) and increased velocities of 27 to 46% (Fig. S6G, 3^rd^ and 5^th^ scatter plots). No significant impact was observed on the directionality of MCF7 cells (Fig. S6H).

MDA-MB-231 exhibited a significantly higher motility rate, likely due to their invasive mesenchymal phenotype. Non-treated and control medium treated MDA-MB-231 cells demonstrated an accumulated distance of 610 μm and 611 μm, respectively (Fig. S6I and J, 1^st^ and 2^nd^ scatter plots). Notably, the supernatants from ln- as well as from ob-aT bASCs significantly increased the accumulated distance and the velocity of MDA-MB-231 cells (Fig. S6I-K), whereas ln- and ob-dT bASCs had no clear effect on these cells (Fig. S6I-K). Like in MCF7 cells, no obvious effect was observed on the directionality of MDA-MB-231 cells (Fig. S6L). Overall, the data suggest that bASCs stimulate the migratory and invasive capacity of malignant cells, as reported for MSCs [25]. In particular, our results indicate that cancer-educated aT bASCs confer a greatly enhanced ability to promote the motility of low- and high-malignant breast cancer cells, majorly due to their increased secretion of bioactive factors including cytokines and chemokines.

### Hybrid spheroids formed with breast cancer cells and bASCs become resistant to tamoxifen and docetaxel

Chemoresistance is a major obstacle in the treatment of breast cancer, and its related molecular mechanisms are not completely understood [10, 51]. To study the roles of bASCs in this aspect, we formed hybrid spheroids consisting of bASCs and low-malignant MCF7^(ER+, PR+, HER2+)^, BT474^(ER+, PR+, HER2+)^ or high-malignant MDA-MB-231^(ER-, PR-, HER2-)^ breast cancer cell lines [27] as displayed in Fig. S7A. These hybrid spheroids were formed with different percentages (85, 70 or 50%, respectively) of breast cancer cells together with 15, 3% or 50% of bASCs. The spheroid area was microscopically measured. MCF7 and MDA-MB-231 breast cancer cells alone showed poor spheroid formation, with an unstructured and unstable phenotype (Fig. S7B-E, 1^st^ scatter plot). The spheroid formation capacity was dramatically increased by adding bASCs (Fig. S7B-E). Hybrid spheroids containing one of the four bASC subgroups significantly increased the spheroid formation, visualized by the area of MCF7 and MDA-MB-231 cells, even with a low concentration of 15% bASCs (Fig. S7B and C, 1^st^ vs. 2^nd^, 5^th^, 8^th^ and 11^th^ scatter plots). This observed effect on the spheroid area was enhanced with increasing concentrations of bASCs (15, 30 and 50%) (Fig. S7B and C). In support of the previous results (Fig. S4 and S5), hybrid spheroids formed with ln-aT or ob-aT bASCs were larger than with the control counterparts at all concentrations used (Fig. S7B and C, dark gray vs. light gray scatter plots). The lowest concentration (15%) was chosen for further experiments to mimic breast cancer subtypes with low stroma content [52].

To analyze the impact of bASCs on cancer cell proliferation, *CellTrace™* tagged MCF7 and MDA-MB-231 spheroids were either cultured with different bASCs supernatants or co-cultured with 15% of bASCs, and the cell count was measured after 96 h by flow cytometry. The control medium did not significantly alter the cell count of MCF7 (Fig. S7F, 1^st^ vs. 2^nd^ boxplot) and MDA-MB-231 cells (Fig. S7G, 1^st^ vs. 2^nd^ boxplot). Moreover, the supernatants of all four bASCs subgroups had no significant impact on the proliferation rate of both cancer cell lines (Fig. S7F and G, 1^st^ to 6^th^ boxplots), except a slight effect of the supernatant from ln-dT bASCs on MDA-MB-231 cells (Fig. S7G, 2^nd^ vs. 4^th^ boxplot). By contrast, MCF7 and MDA-MB-231 spheroids in direct co-culture with bASCs significantly increased their cell numbers (Fig. S7F and G, 2^nd^ vs. 7^th^–10^th^ boxplots). In further support, both cancer cell lines co-cultured with ln-aT or ob-aT bASCs showed the highest proliferation rates compared to cells co-cultured with control medium (Fig. S7F and G, 2^nd^ vs. 7^th^ and 9^th^ boxplots).

**Fig. 5:**
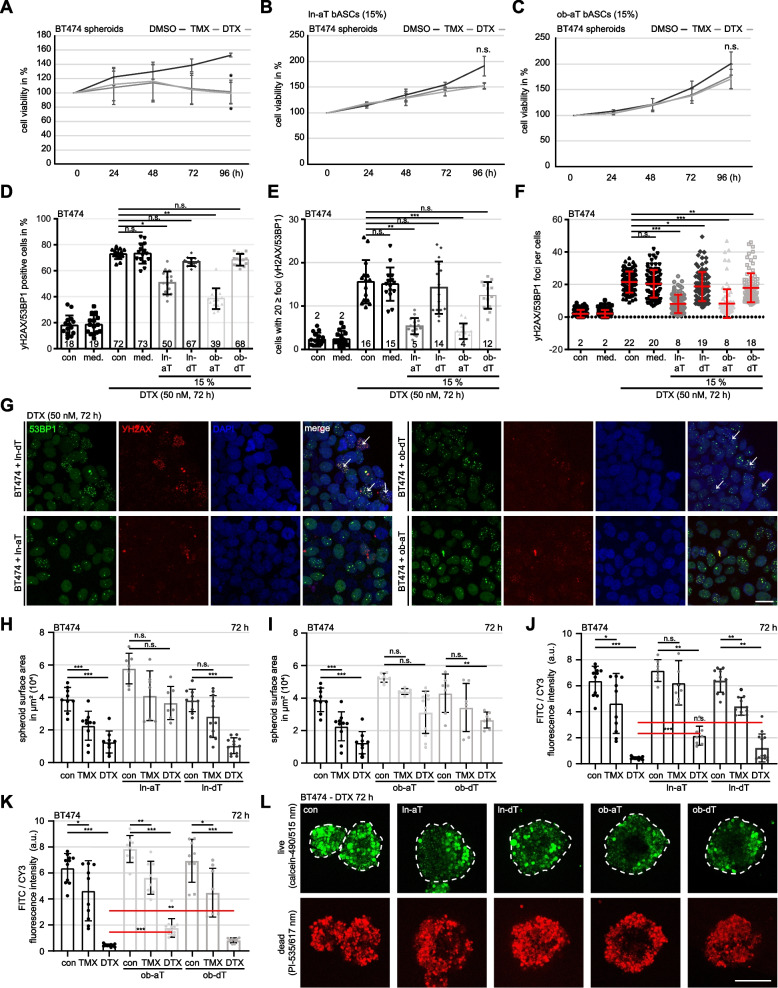
Hybrid spheroids of breast cancer cells and ln-aT/ob-aT bASCs demonstrate an increased resistance to chemotherapeutic agents. **A**-**C** BT474 spheroids (**A**), BT474/ln-aT bASCs (**B**) and BT474/ob-aT bASCs (**C**) hybrid spheroids, generated for 24 h, were transferred into 96-well low attachment plates, treated with DMSO, TMX (5 μM) or DTX (50 nM) for up to 96 h. Cell viability was measured at indicated time points. The results are based on three independent experiments and presented as mean ± SEM. **D**-**G** Indicated spheroids were treated with 50 nM DTX for 72 h, stained for DNA damage markers γ-H2AX (red) and 53BP1 (green), and DNA (DAPI, blue). **G** Representatives are shown. White arrows indicate cell nuclei with 20 ≥ foci. Scale: 25 μm. D**-**F Quantification of γ-H2AX and 53BP1 double positive cells (**D**, *n* = 15 fields in each group), cells with 20 ≥ foci (**E**, *n* = 15 fields in each group) and double positive foci per cell (**F**, *n* = 90 cells in each group). **H**-**L** BT474- and hybrid spheroids were stained using the live/dead viability/cytotoxicity kit. Spheroid surface area (**H** and **I**, *n* = 7–11 fields in each group), and the ratio between the fluorescence intensity of viable cells (calcein/green) and the dead fraction (PI/red) (**J** and **K**, *n* = 7–12 fields in each group) were evaluated. The results are based on three independent experiments and presented as mean ± SEM. **L** Representative images of spheroids treated with DTX (50 nM) for 72 h are shown. White dotted lines depict the measured area of the spheroids. Scale: 250 μm. Student’s t test was used in (**A**-**C**). Unpaired Mann-Whitney U test was used in (**D**-**F**) and (**H**-**K**). *p < 0.05, **p < 0.01, ***p < 0.001

As multiple reports have demonstrated an impact of MSCs on cancer cell chemoresistance [53], we were interested in the potential influence of bASCs on the chemotherapeutic response of breast cancer cells. Hybrid spheroids were formed with BT474 cells (85%) and bASCs (15%) for 72 h (Fig. S7A). After establishing breast cancer cell (BC)/bASC hybrid spheroids, the spheroids were treated up to 96 h with TMX or docetaxel (DTX), two commonly used chemotherapeutic agents for the treatment of breast cancer patients [54], and their proliferation was measured via cell viability assay. BT474 only spheroids showed a significant growth reduction after 96 h (Fig. 5A), whereas BT474 hybrid spheroids formed with ln-aT or ob-aT displayed no significant growth restriction (Fig. 5B and C), indicating an increased resistance toward both chemotherapeutics. By contrast, hybrid spheroids formed with ln-dT and ob-dT bASCs were still sensitive to the treatment with TMX and DTX, as indicated by significantly reduced cell viability compared with control cells treated with DMSO (Fig. S8A and B). These data were further supported by MCF7 and MDA-MB-231 hybrid spheroids. Both cell lines were sensitive to each chemotherapeutic agent (Fig. S8C and H) and lost this sensitivity upon integration of ln-aT or ob-aT bASCs into the spheroids (Fig. S8E, G, J and L). Similar to BT474 cells, MCF7 and MDA-MB-231 spheroids co-cultured with ln-dT and ob-dT bASCs failed to mediate this resistance (Fig. S8D, F, I and K). In conclusion, integration of ln- or ob-aT bASCs prevented apoptosis induction by chemotherapeutic agents in breast cancer spheroids, in contrast to control dT bASCs.

We next analyzed DTX-induced DNA damage and repair in BT474 hybrid spheroids. Spheroid cells were treated with 50 nM of DTX for 72 h, stained for DNA damage markers γ-H2AX (Ser139) and p53-binding protein 1 (53BP1) [55], and three parameters were examined microscopically: double positive cells, cells with 20 or more double positive foci and the overall foci count. As indicated by the growth reduction in the cell viability assay (Fig. 5A), prolonged treatment with DTX induced massive DNA damage in BT474 cells showing a double positive staining in 72–73% of control cells or cells incubated with control medium (Fig. 5D and G, 3^rd^ and 4^th^ bars), 15–16% of cells had 20 or more foci (Fig. 5E, 3^rd^ and 4^th^ bars), and the overall foci count was 20–22 foci per cell (Fig. 5F, 3^rd^ and 4^th^ scatter plots). Both ln- or ob-aT bASC integrated spheroids displayed a similar trend, the number of double positive cells decreased to 39–51% (Fig. 5D and G, 5^th^ and 7^th^ bars), the percentage of cells with ≥20 foci turned down to 4–5% (Fig. 5E, 5^th^ and 7^th^ bars), as well as the foci per cell was reduced to 8–9 (Fig. 5F and G, 5^th^ and 7^th^ scatter plots). The control ln- and ob-dT bASCs were not able to reduce the percentage of positive cells or cells with more than 20 foci in hybrid spheroids (Fig. 5D, E and G, 6^th^ and 8^th^ bars). Only the absolute number of foci was slightly decreased to 18–19 per cell (Fig. 5F, 6^th^ and 8^th^ scatter plots). To further corroborate these data, BT474 hybrid spheroids were labelled with a live (calcein-490 nm/515 nm)/dead (propidium iodide-535 nm/617 nm) marker and treated with TMX (5 μM) or DTX (50 nM) for up to 3 days. The cell surface area and the ratio between live/dead fluorescent signals were analyzed microscopically. In control cells, both chemotherapeutic agents induced apoptosis, indicated by a robust increase in the dead fraction of cells and a decreased cell surface area (Fig. 5H-L, 1^st^ to 3^rd^ bars). Interestingly, BT474 cells, which were reported to display moderate resistance to TMX [56], showed indeed a modest apoptosis induction compared to DTX-treated cells (Fig. 5H-L, 2^nd^ vs. 3^rd^ bars). Consistent with these observations, the cell surface area of BT474/ln/ob-aT bASC hybrid spheroids was not significantly reduced after TMX or DTX treatment (Fig. 5H, I and L) and had a lower live/dead ratio compared with BT474 control cells (Fig. 5J, K and L). In contrast, both ln/ob-dT bASCs only marginally reduced apoptosis induction, as evidenced by slightly decreased fluorescent intensity of propidium iodide and reduced cell surface area (Fig. 5H-L). Taken together, these results imply the potential clinical significance of bASCs in inducing chemoresistance in breast cancer cells. These results provide evidence that a high number of aT bASCs in the TME may influence the response to chemotherapy in primary breast cancer.

### Transcriptomic changes in breast cancer cells induced by lean and obese bASCs

**Fig. 6:**
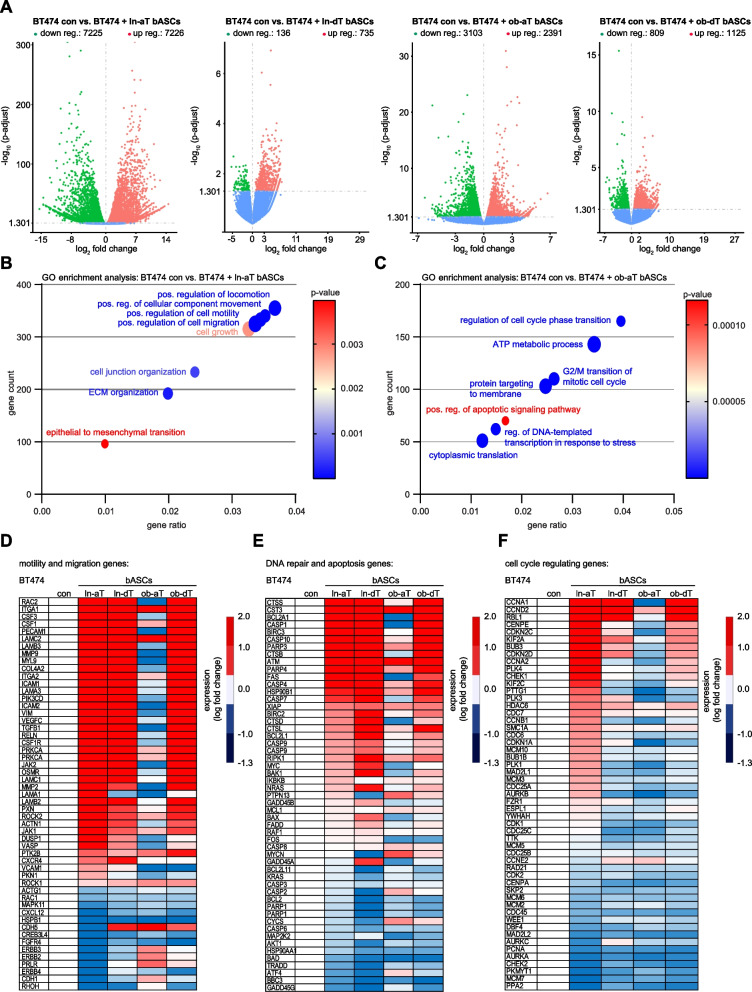
Transcriptome profiles of triple positive BT474 cells directly co-cultured with bASCs. **A**-**F** BT474 cells cultured for 14 days alone or in direct co-culture with different bASCs (ln/ob-dT/aT) were sorted for RNA-seq analysis (*n* = 3). The gene expression was analyzed using DESeq2 R package. **A** Volcano plots show the adjusted *p*-value for genes differentially expressed between control BT474 cells and BT474 cells directly co-cultured with ln-aT bASCs (1^st^ volcano plot), ln-dT bASCs (2^nd^ volcano plot), ob-aT bASCs (3^rd^ volcano plot) and ob-dT bASCs (4^th^ volcano plot). **B** and **C** GO enrichment analysis of RNA-seq data with a *p*-value ≤0.05. Gene count: the number of genes that are deregulated in the pathway. Gene ratio: ratio of the number of target genes divided by the number of all genes in each pathway. Significance is color coded as indicated (high *p*-value, red; low *p*-value, blue). **D**-**F** Heatmaps of deregulated genes involved in motility and migration (**D**), DNA repair and apoptosis (**E**), and cell cycle regulation (**F**). A fold change of ≥2 (red color code) and ≤ − 2 (blue color code) are shown. Included genes have a *p*-adjusted value of ≤0.05 for at least one condition (ln-aT or ob-aT)

To investigate the crosstalk between bASCs and BC cells (BT474 and MDA-MB-231) at the molecular level, both cell types were grown in direct co-culture (85% BCs / 15% bASCs), as in experiments with hybrid spheroids. After 14 days of direct co-culture, the cells were sorted using the classical MSC markers CD90, CD73 and an epithelial marker CD24 (Fig. S8M). Total RNAs were extracted from sorted BC cells for transcriptomic mRNA-sequencing. The RNA-seq data revealed that the direct co-culture with bASCs had a huge impact on both breast cancer cell lines. Co-cultured and sorted BCs had several hundred deregulated (up- and downregulated) genes displayed by volcano plots (Fig. 6A and S9A). Strikingly, co-cultured with ln-aT bASCs induced 16.6-fold more deregulated genes in BT474 cells and 17.8-fold more in MDA-MB-231 cells compared to these cells co-cultured with ln-dT bASCs (Fig. 6A and S9A, 1^st^ vs. 2^nd^ volcano plot). A similar trend of increased up- or downregulated genes could be observed in both BC lines co-cultured with ob-aT bASCs, but not to the same extent as in BC lines co-cultured with ln-aT bASCs (Fig. 6A and S9A, 1^st^ vs. 3^rd^ volcano plot). The GO pathway enrichment analyses revealed that at least three key pathways were affected in BT474 cells by co-culturing with ln-aT bASCs: regulation of cell migration, cell growth and EMT (Fig. 6B). The RNA-seq data for BT474 cells in direct co-culture with ob-aT bASCs were diverse, nevertheless suggesting gene changes in cell cycle progression, cellular metabolism, cellular stress, and apoptosis response (Fig. 6C).

Moreover, heatmaps were generated for the most interesting significant genes involved in cell motility and migration (Fig. 6D), DNA repair and apoptosis (Fig. 6E), and cell cycle regulation (Fig. 6F). Intriguingly, BT474 cells co-cultured with ob-aT bASCs presented an entirely different gene profile (Fig. 6D-F, 4^th^ row), whereas these cells directly co-cultured with ln-aT, ln-dT or ob-dT bASCs had many overlapping genes that were deregulated in the same direction (Fig. 6D-F, 2^nd^, 3^rd^ and 5^th^ rows). This is in accordance with multiple reports highlighting a general tumor-promoting effect of MSCs co-cultured with BC cell lines [57]. The common pattern of breast cancer cells co-cultured with ln-aT, ln-dT or ob-dT bASCs indicated a changed expression of important cytoskeleton genes *RAC2* and *VIM*, ECM genes *LAMC2* and *ICAM1*, DNA repair genes *ATM* and *BIRC2–3,* and cell cycle regulating genes *CCNA1*, *CCND2* and *CENPE* (Fig. 6D-F, 2^nd^, 3^rd^ and 5^th^ row), allowing possible molecular explanations for the increased cell proliferation, motility and chemoresistance observed in the experiments (Fig. 5 and Fig. S4–8). BT474 cells directly co-cultured with ob-aT bASCs altered their transcriptomic profile to a great extent (Fig. 6D-F, 4^th^ row). These co-cultured cells upregulated ECM related genes such as *ITGA1* and *LAMC2*, but downregulated cytoskeleton associated genes including *RAC2* and *VIM* (Fig. 6D, 4^th^ row), suggesting a completely different molecular mechanism for the observed enhanced cell motility (Fig. S6E-J). Despite downregulation of most DNA repair and apoptosis genes, *ATM* was significantly upregulated (*p* < 0.001), together with *MYCN*, an important DNA damage response gene (Fig. 6E, 4^th^ row) [58]. In line with the increased proliferation induced by direct co-culture with ob-aT bASCs (Fig. S4 and 5), many regulatory genes of the cell cycle checkpoint were downregulated, including *BUB3*, *CHEK1–2* and *MAD2L1–2* (Fig. 6F, 4^th^ row) [59].

MDA-MB-231 cells directly co-cultured with ln-aT bASCs displayed deregulated genes in crucial signaling pathways including *PI3K*, *MAPK*, *RAS*, *mTOR*, *TNF*, *ERBB* and *VEGF* (Fig. S9B). Whereas MDA-MB-231 cells co-cultured with ob-aT bASCs affected signaling pathways involved in the cell cycle, apoptosis, DNA repair and TGFβ signaling (Fig. S9C). Distinct from BT474 cells, MDA-MB-231 co-cultured with each of four bASC subgroups presented an upregulated gene expression of various cell cycle regulators such as *CCNE2*, *WEE1*, *AURKA*, consistent with the proliferation data (Fig. S9D). MDA-MB-231 cells co-cultured with ln-aT bASCs had a decreased expression of multiple inflammatory and apoptotic genes such as *CASP1*, CASP4–5 and *CASP7–10,* and an increased expression of various cell survival genes including *MYCN*, *KRAS* and *BCL2* (Fig. S9E, 2^nd^ row). By contrast, MDA-MB-231 cells co-cultured with ob-aT bASCs did not affect the mentioned caspases, but enhanced the gene expression of c-*MYC*, *FOS*, *BCL2* and *BCL2A1* (Fig. S9E, 4^th^ row). Cancer stem cells (CSC) are often associated with an increased drug resistance [60]. To look at this issue, a heatmap was generated with genes concerning stemness in MDA-MB-231 cells (Fig. S9F). The genes of stem cell markers such as *ALDH2*, *BCL2*, *WEE1*, *TP63* and *SOX2* were upregulated in these cells co-cultured with each of aT bASCs (Fig. S9F, 2^nd^ and 4^th^ rows). Additionally, MDA-MB-231 cells co-cultured with ob-aT bASCs also overexpressed *ALDH1A1* and *KLF4* (Fig. S9F, 4^th^ row). In conclusion, direct co-culture with aT bASCs had a major impact on the transcriptomic profile of low- as well as high-malignant breast cancer cells by greatly altering the expression of crucial genes associated with the cell cycle, DNA repair and motility.

### Both aT bASC subtypes trigger the EMT in epithelial breast cancer cells and increase cancer stem cell markers in mesenchymal breast cancer cells

**Fig. 7:**
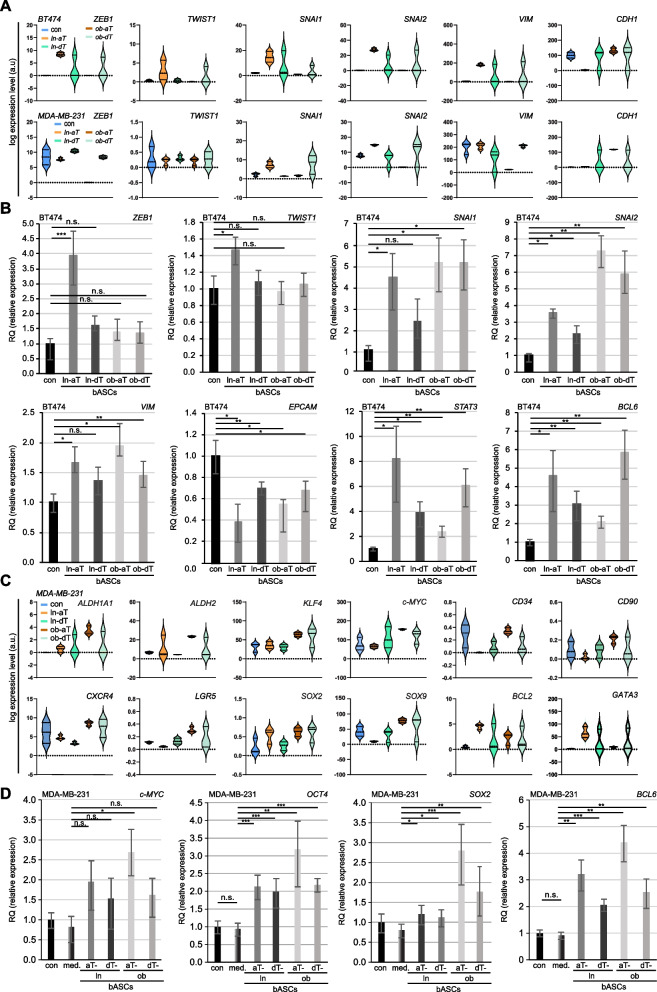
ln-aT bASCs induce the EMT in BT474 cells, whereas ob-aT bASCs stimulate MDA-MB-231 cells into a CSC phenotype. **A** and **C** BT474 and MDA-MB-231 cells were directly co-cultured with different bASCs (ln-aT, ln-dT, ob-aT and ob-dT) and were sorted for RNA-seq. Three independent experiments were performed for this analysis. **A** Representative violin plots show selected EMT marker genes. Values represent the log expression levels of genes from the RNA-seq data. Included genes have an adjusted *p*-value of ≤0.05 for at least one condition (ln-aT or ob-aT). **B** Relative levels of EMT-associated genes, including *TWIST1, ZEB1*, *SNAI1*, *SNAI2*, *VIM*, *EPCAM*, *STAT3* and *BCL6*, are shown for different bASC subgroups (ln-dT, ln-aT, ob-dT and ob-aT). The results are from three independent experiments and presented as bar graphs with mean ± SEM. **C** Representative violin plots show selected CSC marker genes. Values represent the log expression levels of genes from the RNA-seq data. Included genes have an adjusted *p*-value of ≤0.05 for at least one condition (ln-aT or ob-aT). **D** Relative gene levels of CSC-associated genes c-*MYC*, *OCT4*, *SOX2* and *BCL6* are shown for different bASC subgroups (ln-dT, ln-aT, ob-dT and ob-aT). The results are from three independent experiments and presented as bar graphs with mean ± SEM. Student’s t test was used. *p < 0.05, **p < 0.01, ***p < 0.001

The involvement of the EMT in drug resistance is well documented both in vitro as well as in vivo [61]. To address this, the deregulated EMT related genes (Fig. 6B) were further analyzed. As depicted by the violin plots, BT474 cells co-cultured with ln-aT bASCs highly upregulated the gene expression of EMT transcriptional factors including *ZEB1*, *TWIST1*, *SNAI1* and *SNAI2*, with an associated increase of *VIM* as well as a decrease of *CDH1* (Fig. 7A, 2^nd^ violin plot). Though in a similar pattern, BT474 cells co-cultured with ob-dT bASCs displayed a reduced extent compared to ln-aT bASCs (Fig. 7A, 2^nd^, 3^rd^ and 5^th^ violin plots). Co-culture with ob-aT bASCs had hardly an effect on EMT related genes in BT474 cells (Fig. 7A, 4^th^ violin plot). Intriguingly, the mesenchymal MDA-MB-231 cells co-cultured with bASCs displayed minor changes in EMT-related genes and co-culture of ob-aT bASCs even downregulated gene levels of *ZEB1*, *SNAI1*, *SNAI2* and *VIM*, together with an increased gene expression of *CDH1* (Fig. 7A, 1^st^ vs. 4^th^ violin plot). To corroborate these RNA-seq data, we performed qPCR analyses with sorted BT474 cells directly co-cultured with different bASCs for 14 days. Indeed, the qPCR results demonstrated an EMT induction in the BT474 cells (Fig. 7B). The ln-aT bASCs affected significantly the EMT related genes by upregulating *ZEB1*, *TWIST1*, *SNAI1*, *SNAI2* and *VIM* (Fig. 7B, graph 1 to 5, 2^nd^ rows). *EPCAM*, an important epithelial marker, was downregulated in these cells (Fig. 7B, graph 6, 2^nd^ row). In addition, two other genes indirectly involved in the EMT, *STAT3* as well as *BCL6,* were also upregulated (Fig. 7B, graph 7 and 8, 2^nd^ row). Interestingly, BT474 cells co-cultured with each of the subgroups (ln/ob-dT and ob-aT) presented a similar tendency with increased gene expression of *SNAI1, SNAI2, VIM*, *STAT3* and *BCL6,* and a decreased level of *ECPAM* (Fig. 7B).

The combined mRNA data from qPCR and RNA-seq were further corroborated with immunofluorescence staining of E-cadherin, an epithelial marker, and N-cadherin, a mesenchymal marker, in BT474 and MCF7 cells indirectly co-cultured with bASCs subgroups. The results showed that ln-aT bASCs were able to significantly increase the size of MCF7 and BT474 cells (Fig. S10A, B and E), which is a direct consequence of reduced apical-basal polarity. In line with this observation, the co-culture with ln-aT bASCs reduced the E-cadherin signal, whereas the N-cadherin signal was drastically increased (Fig. S10A-G). All other bASC subgroups (ln-dT, ob-dT and ob-aT) were able to partly induce EMT in MCF7 and BT474 cells, but to a less extent compared to ln-aT bASCs (Fig. S10A-D). These results underscore the notion that ln-aT bASCs are the most potent subgroup to induce the EMT in breast cancer cells (Fig. S10A-G).

MDA-MB-231 cells co-cultured with bASCs exhibited barely any change in the expression of EMT-related genes (Fig. 7A, lower panel). Instead, as shown in the heatmap (Fig. S9F), multiple CSC-associated genes were affected in the transcriptome data (Fig. 7C). These included the upregulation of classical CSC genes *c-MYC*, *ALDH1A1*, *ALDH2*, *KLF4, CD34, CD90, CXCR5* and *LGR5* [62], as well as genes involved in pluripotency including *SOX2*, *SOX9*, *BCL2* and *GATA3* (Fig. 7C). To corroborate these data, we performed qPCR with the same sorted MDA-MB-231 cells. These cells demonstrated indeed significantly increased *NANOG, OCT4, SOX2, SOX9* and *BCL6*, moderately elevated c-*MYC* and *KLF4*, and reduced *BCL2* gene levels (Fig. 7D and Fig. S10H). Particularly, ob-aT bASCs triggered the CSC gene expression more strongly than ln-aT bASCs in MDA-MB-231 cells (Fig. 7D and Fig. S10H, 3^rd^ vs. 5^th^ bar). In sum, the results highlight an immense impact of bASCs on the transcriptome profile of breast cancer cells. Specifically, ob-aT bASCs induce a more CSC-like phenotype in highly malignant mesenchymal MDA-MB-231 cells.

### The expression of LIF and αSMA in the cancer tissue stroma correlates with BMI of breast cancer patients

**Fig. 8:**
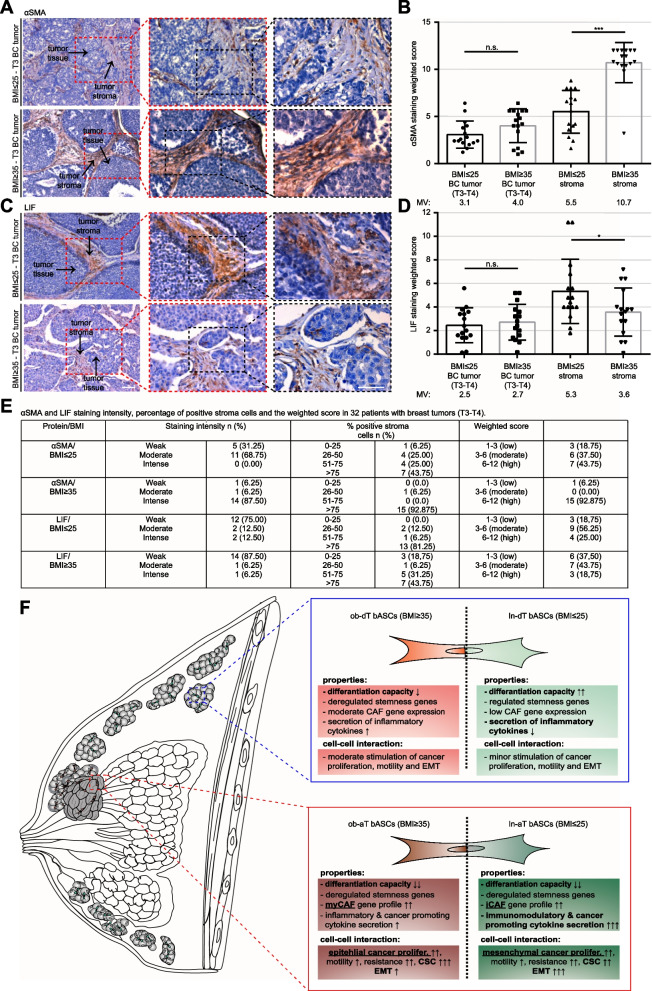
Increased αSMA and LIF in the cancer stroma from obese and lean patients, respectively. **A** and **C** Formalin-fixed and paraffin-embedded (FFPE) T3/4 breast cancer tissue sections of lean (BMI ≤ 25) or obese (BMI ≥ 35) patients were immunohistochemically stained with LIF (**C**) or αSMA (**A**) antibody (brown), respectively, and counterstained with hematoxylin (blue). Scale: 100 μm. Inset I and II scale: 50 μm. **B** and **D** Quantification of the weighted score in breast cancer cells and tumor stroma for LIF (**D**) and αSMA (**B**). The results are presented as bar and scatter plots showing the mean value ± SEM (*n* = 32, 16 lean / 16 obese breast cancer tissues). Student’s t test was used. *p < 0.05, ***p < 0.001. **E** Table depicts LIF and αSMA staining intensity, percentage of positive tumor stroma and weighted score in 32 breast cancer tissue sections. **F** Schematic illustration of the proposed working model. The breast cancer TME has a crucial impact on the properties of bASCs and on their cell-cell interaction capacity. The de-differentiation process within the TME is highly dependent on the cellular context and is influenced by BMI of patients. Both cancer-educated bASCs phenotypes, iCAF and myCAF, have drastic impacts on breast cancer cells, leading to different tumor promoting profiles in low and high malignant cancer cells

To address if BMI of the breast cancer patients could be related to the de-differentiation processes in the breast cancer tissue, as indicated in Table S4, 32 breast cancer samples classified as T3/T4 from 16 lean- (BMI ≤ 25) as well as from 16 patients with obesity (BMI ≥ 35) were stained for LIF (iCAF) and αSMA (myCAF) by immunochemistry and evaluated with the weighted score [63]. Although both staining showed no significant changes in the breast cancer cell population (Fig. 8A-E), the tumor stroma displayed a significant correlation between BMI and both stained proteins (Fig. 8A-E). The weighted score for the αSMA staining was increased from 5.5 in lean patients to 10.7 in obese patients (Fig. 8A, B and E). On the contrary, the LIF staining was increased in lean patients with 5.3 compared to 3.6 in patients with obesity (Fig. 8C-E). These data point to the notion that the breast cancer stroma is significantly affected by BMI of breast cancer patients, with crucial consequences for the phenotype and expression profile of the TME cells including bASCs.

## Discussion

Despite intense research, questions remain regarding the relationship and interaction between MSCs/ASCs and malignant cells. There are various reports suggesting tumor-promoting effects on the one hand and tumor-inhibiting functions on the other hand [53, 57, 64]. In the present work, we show close reciprocal exchange between bASCs of the TME and breast cancer cells. This crosstalk leads to transcriptomic changes in bASCs that in turn fuel malignancy of breast cancer cells. These findings are achieved by employing bASCs from mammary adipose tissue distant and adjacent to breast cancers of the same patient, distinguishing this study from other investigations that used MSCs of non-mammary origins [20, 65], only tumor adjacent cells [66, 67] or “cancer-educated” MSCs [68]. The observed de-differentiation of bASCs is of particular significance. bASCs near breast cancers from lean patients adopt an inflammatory cancer-educated phenotype, whereas obesity mainly leads to the development of a myofibroblastic cancer-associated phenotype in vitro and ex vivo. By cytokine/chemokine secretion and direct cell-cell contact, both cancer-educated bASCs closely communicate with breast cancer cells and other components of the TME, thus reinforcing the malignancy of breast cancer.

We show that aT bASCs significantly reduce their ability to differentiate into all three aforementioned lineages, in accordance with a previous study reporting a reduced adipogenic differentiation capacity of ASCs from the breast cancer TME [69]. RNA-seq data provide several possible molecular explanations. First, compared to their counterparts ln-dT bASCs, 219 differentiation-related genes were significantly deregulated in ln-aT bASCs including the upregulated genes *ERBB4*, *ROBO2* and *SLIT2*. *ERBB4* is known to promote proliferation in aged MSCs via activating the PI3K/AKT and MAPK pathways [70]. The overexpression of secreted glycoprotein ligand SLIT2 and its immunoglobulin receptor ROBO2 are associated with reduced osteogenic differentiation in rat and murine mesenchymal progenitor cells [71]. Second, the transcription factor *E2F1*, known for its essential role of controlling stem cell fate by regulating the transcription of key cell cycle regulators cyclin A/D/E in various stem cell lineages [72, 73], is upregulated in ln-aT. Indeed, the genes *CCND1*, *CCND3* and *CCNE1* were significantly upregulated in ln-aT bASCs compared to their distant counterparts. Of importance, like ln-aT bASCs, ob-aT as well as ob-dT bASCs display enhanced *E2F1*. ob-dT bASCs differentiate less efficiently than ln-dT bASC, strengthening the negative impact of obesity on the differentiation capacity of MSCs in various adipose tissue types [9, 74]. These data highly suggest that the TME renders bASCs to a more actively proliferating phenotype, whereas genes and pathways responsible for differentiation are downregulated.

Another explanation for the reduced differentiation capacity is the de-differentiation of bASCs into cancer-associated phenotypes, demonstrated by the changed transcriptome profiles, which mostly resemble CAF-like signatures in the TME [38]. Recent approaches such as RNA-seq, proteomics and single-cell sequencing have revealed several CAF subtypes with specific gene signatures and specialized tumor promoting functions [16, 38, 47]. The two most prominent populations are myCAFs and iCAFs. While myCAFs, characterized by high expression of *αSMA*, *TAGLN*, *CTGF* and *FAP*, are involved in ECM remodeling, promoting cancer cell migration, invasion and therapy resistance [40, 75, 76], iCAFs, classified by expression of cytokine genes including *LIF*, *IL1β*, *CSF3*, *CXCL10* and *CCL2*, exert immunosuppressive and tumor growth promoting functions in the TME [38, 40, 77]. Interestingly, ln-aT bASCs more closely resemble an iCAF phenotype associated with high secretion of immunomodulatory cytokines, whereas ob-aT bASCs are more similar to myCAFs, underscoring a significant impact of obesity on bASC de-differentiation. These findings are in accordance with a previous study showing that ASCs from obese patients were more prone to take over a myCAF-like phenotype after direct co-culture with low-malignant cancer cells [78]. This could be explained by chronic inflammation and increased inflammatory cytokines in the obese state, which are known to “activate” fibroblasts and stimulate the expression of αSMA [78, 79]. These data provide evidence that the TME is capable of reducing the differentiation capability of bASCs and instead ob-aT bASCs and ln-aT bASCs into cancer-educated myCAFs and iCAFs, respectively, which further fuel cancer progression via different molecular signaling pathways.

Despite the differences observed in the RNA-seq analyses, both ln- and ob-aT bASCs significantly increase proliferation, spheroid formation, and motility of breast cancer cells. Surprisingly, compared to ob-aT bASCs, ln-aT bASCs are less efficient in stimulating the proliferation of epithelial-like breast cancer cell lines BT474, MCF7 and MDA-MB-361, defying their secretion of cytokines such as FGF/FGF2, CSF3, CXCL10 and LIF. This could be explained by a strong induction of the EMT, known to gradually reduce cell proliferation [80]. In line with this notion, the mesenchymal MDA-MB-231 cells [27] showed an increased proliferation upon co-culture with ln-aT bASCs compared to ob-aT bASCs. Moreover, obese bASCs have an increased secretion of IL6, which has been shown to trigger cancer cell proliferation by activating the JAK/STAT3, ERK1/2 and STAT3/NFĸB pathways in multiple cancer entities including breast cancer [81]. Finally, it was shown that myCAFs were located in a direct proximity to pancreatic tumors, whereas iCAFs were found far from neoplastic cells [82]. This suggests that ob-aT bASCs may crosstalk to breast cancer cells directly via cell-cell contact or ECM remodeling, whereas ln-aT bASCs communicate to breast cancer cells indirectly via secreted cytokines/chemokines. This assumption is further strengthened by a report showing that the direct co-culture of myCAFs increased the proliferation of various pancreatic epithelial cancer cell lines [83]. Thus, ob-aT bASCs are more capable of promoting proliferation in epithelial breast cancer cells as well as activating CSC-associated genes in mesenchymal breast cancer cells. This might help to explain, at least partially, why obesity is associated with reduced survival of postmenopausal breast cancer patients, with an increased mesenchymal/mesenchymal stem cell-like origin, and progressive cancer of premenopausal breast cancer patients with a more epithelial type [84].

CAFs, independent of their subtype, have also been described to increase tumor chemoresistance in vivo and in vitro [85, 86]. Among these different CAF subtypes, iCAFs are reported to have a particular effect on chemoresistance in breast cancer patients [40, 86], probably due to their high secretion of cytokines involved in therapy resistance, such as CXCL1–3, CCL2, CSF3, CXCL10, IL1β and LIF. These cytokines have been shown to play critical roles in regulating tumor immune tolerance [87], recruitment and de-differentiation of myeloid-derived suppressor cells [86], EMT induction [88], activation of STAT3, PI3K, MAPK and Rho signaling pathways [89], and inducing cancer stemness in breast cancer cells [90]. Indeed, ln-aT bASCs were highly effective in triggering the EMT in low-malignant BT474 cells involved in the development of chemoresistance [91] through the activation of multiple known EMT-inducing pathways, including JAK/STAT3/PI3K/TGFβ [92], as shown in the transcriptome and qPCR analysis. ob-aT bASCs exhibited an increased ability to stimulate cancer stemness in highly malignant MDA-MB-231 cells by enhancing the gene expression of pluripotency and CSC associated genes such as *ALDH1H1*, *ALDH2*, c-*MYC*, CD34, LGR5, CXCR4, *SOX2* and *OCT4* [62, 93], fueling further their malignant potential. Moreover, both ln/ob-aT bASCs stimulate the gene expression of ATM, an essential component of the DNA double-strand break repair mechanism, which may explain reduced DNA foci number and decreased apoptotic response in breast cancer cells treated with DTX, since the combination of ATM and ATR inhibitors is already used to re-sensitize breast cancer cells in clinical trials [94]. Together, bASCs may exert multiple mechanisms to increase therapy resistance, in particular, their de-differentiation into CAFs triggers EMT induction, stemness generation and increased cell survival of breast cancer cells. More investigations are warranted to study the detailed impact of ln/ob-aT bASCs on breast cancer chemoresistance and their immunomodulatory function in vitro and in vivo.

Finally, an ex vivo correlation has been established between the expression of iCAF marker LIF and myCAF marker αSMA with breast cancer tissue sections from patients with or without obesity. Specifically, the tissue sections of obese patients displayed a significantly higher αSMA level in stroma compared to the sections from lean patients. This is of specific importance, as it has been recently reported that the stromal expression of αSMA is highly associated with the resistance to trastuzumab in patients with early-stage Her2 positive breast cancer [95]. Additionally, the expression of αSMA was found to be correlated with poor prognosis in patients with ER positive breast cancer [96] and in patients with luminal breast cancer [97], likely mediated by a TMX resistance and an increased proliferation. Moreover, the expression of LIF in the stroma was significantly elevated in breast cancer tissue sections from lean patients. Interestingly, the high expression of LIF has been connected to increased tumor progression, stemness, therapy resistance and TME modulation in various cancer entities in multiple in vitro and in vivo models [98] and the immunization against LIF or LIFR suppressed the tumor formation, growth and metastasis in a mammary xenograft mouse model [99]. Our findings clearly suggest that obesity influences the TME. These insights could be of great significance in determining the fate of breast cancer development and be useful for future targeted therapies.

## Conclusion

This work demonstrates the different de-differentiation processes of bASCs in the TME of breast cancers from obese and lean patients, relative to their counterparts from cancer distant adipose tissues of the same patients to exclude individual specific features. Importantly, ln/ob-aT bASCs greatly reduce their differentiation capacity. Instead, ln-aT bASCs and ob-aT bASCs alter their transcriptome as well as secretome toward an iCAF or myCAF profile, respectively. These cancer-educated bASCs are connected to increased proliferation, motility and chemoresistance of breast cancer cell spheroids. Intriguingly, ln-aT bASCs induce the EMT in epithelial-like breast cancer cells, whereas ob-aT bASCs are more potent to enrich CSC genes in mesenchymal-like breast cancer cells (Fig. 8F), contributing further to chemotherapy resistance. Additional studies are required to corroborate these findings and their in vivo significance. It is also important to explore the precise molecular mechanisms by which the TME affects the function of bASCs in an obesity-dependent manner, and how cancer-educated bASCs fuel breast cancer progression at molecular levels.

## Supplementary Information

**Additional file 1: Supplementary information. Supplementary Table 1.** Clinical information of breast cancer patients, whose mammary adipose tissues were used for bASC isolation. **Supplementary Table 2.** Cell surface markers of different lean bASCs. **Supplementary Table 3.** Cell surface markers of different obese bASCs. **Supplementary Table 4.** Clinical information of breast cancer patients, whose mammary adipose tissues were used for IHC staining. **Figure S1.** No obvious alterations in cell cycle distribution and cell viability among various bASC subgroups, and more COLA1 and vimentin in aT-bASCs than in dT-bASCs. **Figure S2.** Both ln- and ob-aT bASCs display deregulated differentiated associated genes and ln-aT bASCs increased iCAF genes. **Figure S3.** Secretion of bASCs alone or upon incubation with MDA-MB-231 cells. **Figure S4**. ob- and ln-aT bASCs promote the growth of BT474 spheroids. **Figure S5.** Direct co-culture of ob- and ln-aT bASCs promotes the growth of BT474 and MDA-MB-361 spheroids. **Figure S6:** Ob- and ln-aT bASCs promote proliferation of MDA-MB-361 spheroids and their supernatants enhance the motility of breast cancer cells. **Figure S7.** bASCs promote the formation and growth of spheroids of breast cancer cells. **Figure S8.** Hybrid spheroids of breast cancer cells and bASCs demonstrate an increased resistance to chemotherapeutic agents. **Figure S9.** Transcriptome profiles of triple negative MDA-MB-231 cells directly co-cultured with bASCs. **Figure S10.** ln-aT bASCs efficiently trigger EMT in epithelial breast cancer cell lines, while ob-aT bASCs more efficiently stimulate MDA-MB-231 cells into a CSC phenotype.

## Data Availability

All data generated or analyzed during this study are included in this article and its supplementary information.
