# Supplementary material for: Cancer-educated mammary adipose tissue-derived stromal/stem cells in obesity and breast cancer: spatial regulation and function
Source: J Exp Clin Cancer Res. 2023 Jan 30;42:35. doi: 10.1186/s13046-022-02592-y (PMC9885659; doi:10.1186/s13046-022-02592-y)
Supplement: Supplementary file 1 — Additional file 1: Supplementary information. Supplementary Table 1. Clinical information of breast cancer patients, whose mammary adipose tissues were used for bASC isolation. Supplementary Table 2. Cell surface markers of different lean bASCs. Supplementary Table 3. Cell surface markers of different obese bASCs. Supplementary Table 4. Clinical information of breast cancer patients, whose mammary adipose tissues were used for IHC staining. Figure S1. No obvious alterations in cell cycle distribution and cell viability among various bASC subgroups, and more COLA1 and vimentin in aT-bASCs than in dT-bASCs. Figure S2. Both ln- and ob-aT bASCs display deregulated differentiated associated genes and ln-aT bASCs increased iCAF genes. Figure S3. Secretion of bASCs alone or upon incubation with MDA-MB-231 cells. Figure S4. ob- and ln-aT bASCs promote the growth of BT474 spheroids. Figure S5. Direct co-culture of ob- and ln-aT bASCs promotes the growth of BT474 and MDA-MB-361 spheroids. Figure S6: Ob- and ln-aT bASCs promote proliferation of MDA-MB-361 spheroids and their supernatants enhance the motility of breast cancer cells. Figure S7. bASCs promote the formation and growth of spheroids of breast cancer cells. Figure S8. Hybrid spheroids of breast cancer cells and bASCs demonstrate an increased resistance to chemotherapeutic agents. Figure S9. Transcriptome profiles of triple negative MDA-MB-231 cells directly co-cultured with bASCs. Figure S10. ln-aT bASCs efficiently trigger EMT in epithelial breast cancer cell lines, while ob-aT bASCs more efficiently stimulate MDA-MB-231 cells into a CSC phenotype. [file 13046_2022_2592_MOESM1_ESM.docx]

**Online Data Supplement**

**Cancer-educated mammary adipose tissue-derived stromal/stem cells in obesity and breast cancer: spatial regulation and function**

Andreas Ritter^1^, Nina-Naomi Kreis, Susanne Roth, Alexandra Friemel, Babek Kahn Safdar,

Samira Catharina Hoock, Julia Maria Wildner, Roman Allert,

Frank Louwen, Christine Solbach* and Juping Yuan^1^*

^Obstetrics and Prenatal Medicine, Gynecology and Obstetrics, University Hospital Frankfurt, J. W. Goethe-University, Theodor-Stern-Kai 7, D-60590 Frankfurt, Germany^

^* equal as last authors^

^1 Correspondence: Andreas.Ritter@kgu.de and Yuan@em.uni-frankfurt.de; Tel.: +49-069-6031-83297 (A.R.)^

Running title: Impact of lean and obese adipose tissue-derived mesenchymal stromal/stem cells on breast cancer cells

**Corresponding Authors:**

Dr. Andreas Ritter: Email: andreas.ritter@kgu.de; Tel.: +49 69 6301 83297

Dr. Juping Yuan: Email: yuan@em.uni-frankfurt.de; Tel.: +49 69 6301 5819

Obstetrics and Prenatal Medicine, Gynecology and Obstetrics, University Hospital Frankfurt, J. W. Goethe-University, Theodor-Stern-Kai 7, D-60590 Frankfurt, Germany

We declare no conflict of interest, both financial and personal.

**1. Methods**

**1.1 Adipogenic-, Osteogenic- and chondrogenic differentiation**

The Adipogenic differentiation of bASCs was induced by using StemMACS^TM^ AdipoDiff Media (Miltenyi Biotec, Gladbach, Germany, #130-091-677) for 14 days. Cells were then fixed and stained for α-tubulin, phalloidin and DAPI to visualize lipid vacuoles. The cells were categorized in non-adipocytes, immature adipocytes (vacuoles < 3 µm) and mature adipocytes (vacuoles > 3 µm) depending on their lipid vacuole size. Further the characteristic genes *PPARγ*, *ADIPOQ* and *LEPTIN* were analyzed. For osteogenic differentiation, ASCs were incubated with StemMACS^TM^ OsteoDiff Media (Miltenyi Biotec, Gladbach, Germany, #130-091-678) for 21 days, fixed and stained with 2% Alizarin Red S (pH 4.2) to visualize calcification in cells of an osteogenic lineage. Cells were analyzed for calcific deposition, the mean gray value of microscopic pictures and marker genes *KLF4*, *PTCH1*, *c-MYC*, *OSTEOPONTIN* and *RUNX2.* Chondrogenic differentiation was induced by incubating bASCs with StemMACS^TM^ ChondroDiff Media (Miltenyi Biotec, Gladbach, Germany, #130 091-679) up to 21 days. Cells were fixed and stained with an Alcian blue solution (Merck, Darmstadt, Germany, #TMS-010-C) to visualize sulfated proteoglycans deposits, indicative of functional chondrocytes and the mean gray value was quantified.

**1.2. Cell viability, cell cycle distribution and flow cytometry (FACS)**

Cell viability was measured via CellTiter-Blue^®^ assay (Promega GmbH, Walldorf, Germany, #G808B) as instructed. For cell cycle evaluation, cells were harvested, washed with PBS, fixed with chilled 70% ethanol for 30 min at 4°C, and treated with 1 mg/ml of RNase A (Sigma-Aldrich, Taufkirchen, Germany, #232-646-6) and stained with 100 μg/ml of propidium iodide (PI) (Sigma-Aldrich, Taufkirchen, Germany, #P1304MP) for 30 min at 37°C. DNA content was determined using a FACSCalibur^TM^ (BD Biosciences, Heidelberg, Germany). To analyze the cell surface marker expression, cells were harvested with 0.25% trypsin, fixed for 15 min with ice-cold 2% PFA at 4°C. Cells were washed twice with FCB buffer (PBS, 0.2% Tween-20, 2% FCS) and stained with the following antibodies from eBioscience (Frankfurt am Main, Germany): FITC-conjugated anti-human CD90, PerCP-Cy5.5-conjugated anti-human CD90, PE-conjugated anti-human CD14, FITC-conjugated anti-human CD34, PerCP-Cy5.5-conjugated anti-human CD146, FITC-conjugated anti-human CD105, APC-conjugated anti-human CD106 and APC-conjugated anti-human CD31, PE-conjugated anti-human FSP1, FITC-conjugated anti-human αSMA, APC-conjugated anti-human CD29. Additionally, following antibodies were purchased from BD Pharmingen^TM^ (Heidelberg, Germany): PE-conjugated anti-human CD73, FITC-conjugated anti-human CD14 and APC-conjugated anti-human CD106. To measure FSP1 and αSMA, 0.2% Triton^TM^ X-100 (Sigma-Aldrich, Taufkirchen, Germany, #T8787) was added into the ice-cold 2% PFA to permeabilize the cell membrane.

**1.3 Indirect immunofluorescence staining, immunohistochemistry of breast cancer tissue, imaging and signal intensity measurement**

For indirect immunofluorescence (IF) staining, cells were seeded on Nunc^TM^ Lab-Tek^TM^ II chamber slides (Thermo Fisher Scientific, Waltham, USA, #C6807-1CS) and fixed with 4% paraformaldehyde containing 0.2% Triton^TM^ X-100 (Sigma-Aldrich, Taufkirchen, Germany, #T8787) for 15 min at room temperature. The following primary antibodies were used: mouse monoclonal antibody against vimentin (DAKO, Frankfurt am Main, Germany, #M7020), mouse monoclonal anti-phospho-histone γ-H2AX (Ser139) (Merck Millipore, Darmstadt, Germany, #05-636), polyclonal rabbit antibodies against 53BP1 (Novus, Cambridge, UK, #NB100-304), rabbit polyclonal antibody against phospho-histone H3 (pHH3, Ser10) (Cell Signaling, Frankfurt, Germany, #9701), rabbit polyclonal antibody against αSMA (Abcam, Cambridge, UK, #ab5694) and rabbit monoclonal antibody against COL1A1 (Cell Signaling, Frankfurt, Germany, #E8I9Z). FITC‐, Cy3‐, and Cy5‐conjugated secondary antibodies were obtained from Jackson Immunoresearch (Cambridgeshire, UK). DNA was stained with DAPI (4′,6-diamidino-2-phenylindole-dihydrochloride, Roche, Mannheim, Germany). The actin cytoskeleton was stained by using phalloidin-TRITC (Sigma-Aldrich, Taufkirchen, Germany, #P1951).

A standard staining procedure with DAKO EnVision^TM^FLEX Kit (#K8000; DAKO, Hamburg, Germany) was used to stain formalin-fixed and paraffin-embedded breast cancer tissue sections from breast cancer patients. The following antibodies were used: rabbit polyclonal antibody against LIF (#PA5-79600; 1:50, incubation 1 h at 37 °C, Thermo Fisher Scientific, Waltham, USA) and rabbit polyclonal antibody against αSMA (#ab5694; 1:50, incubation 1 h, Abcam, Cambridge, UK). Slides were counterstained with hematoxylin and analyzed using an AxioObserver.Z1 microscope (Zeiss, Göttingen, Germany). Negative controls included samples stained with control immunoglobulin G (IgG) lacking primary antibody. Evaluation was carried out without knowing the patient data. The average of the percentage of positive cells was determined. Five fields per sample were counted. The slides were further evaluated by the semi-quantitative weighted score method, which takes the staining intensity and the percentage of stained cells into account. The weighted score is determined by adding the results of multiplying the percentage of positive stained cells (0-25 % (1), 26-50 (2), 51-75 (3) and >75 (4)) with their staining intensity (scoring: 1 = weak, 2 = moderate and 3 = strong).

Slides were examined using an AxioObserver (Zeiss). Z1 microscope and images were taken using an AxioCam MRm camera (Zeiss). The slides were further examined by confocal laser scanning microscopy (CLSM) using Z-stack images with a HCXPI APO CS 63.0 × 1.4 oil objective (Leica CTR 6500, Heidelberg, Germany). A series of Z-stack images were captured at 0.5 μm intervals for overlays (superimposing individual images from confocal Z-sections). Representatives are generated by superimposing (overlay) individual images from confocal Z-sections.

Fluorescence intensity was analyzed using line-scan-based analysis via ImageJ (National Institutes of Health). The mean values of fluorescence signals were obtained for each group and were plotted to GraphPad Prism 9 (GraphPad Software Inc., San Diego, USA).

**1.4 Western blot analysis**

For Western blot (WB) analysis, cells were lysed with RIPA buffer (50 mM Tris pH 8.0, 150 mM NaCl, 1% NP-40, 0.5% Na-desoxycholate, 0.1% SDS, 1 mM NaF, phosphatase and protease inhibitor cocktail tablets (Roche, Mannheim, Germany)). The following antibodies were used: mouse monoclonal antibody against GAPDH (GeneTex, Eching, Germany, #GTX627408), mouse monoclonal antibody against β-actin (Sigma-Aldrich, Taufkirchen, Germany, #a2228), rabbit polyclonal antibody against αSMA (Abcam, Cambridge, UK, #ab5694), rabbit monoclonal antibody against COL1A1 (Cell Signaling, Frankfurt, Germany, #E8I9Z), rabbit monoclonal antibody against AKT1 (Cell Signaling, Frankfurt, Germany, #D9R8K), rabbit monoclonal antibody against pAKT (Ser473) (Cell Signaling, Frankfurt, Germany, #D9E), rabbit monoclonal antibody against pGSK3β (Ser9) (Cell Signaling, Frankfurt, Germany, #D85E12) and rabbit monoclonal antibody against caveolin-1 (Cell Signaling, Frankfurt, Germany, # D46G3). ImageJ 1.48v software (National Institutes of Health, Bethesda, MD, USA) was used for densitometric measurements of Western blot analysis.

**1.5 RNA extraction, real-time PCR (qPCR) and transcriptomic analysis (RNA-seq)**

Total RNAs of bASCs, BT474, MDA-MB-231 and MCF7 were extracted with EXTRACTME Total RNA Kit (7Bioscience GmbH, Neuenburg am Rhein, Germany). Reverse transcription was performed using GoScript™ Reverse Transcription Mix, Random Primers (Promega GmbH, Walldorf, Germany). Real-time PCR was performed with a StepOnePlus Real-time PCR System (Applied Biosystems). The data were analyzed using StepOne Software v.2.3 (Applied Biosystems) and QuantStudio^TM^ v.15.2. (Applied Biosystems). Using the comparative CT method, the gene expression was represented as ΔCT, which is normalized to GAPDH as endogenous control and is inversely related to the amount of target molecules in the reaction. The final results were represented as relative quantification (RQ), indicating the difference in gene expression level between the analyzed samples, by setting the expression value of control condition as 1 in mean with minimum and maximum range. The RQ of a group is defined as 2^−(ΔCTgroup−ΔCTcontrol)^.

All probes for gene analysis were obtained from Applied Biosystems: *ALDH1H1* (#Hs00946916_m1), *ALDH2* (#Hs01007998_m1), *ACTA2* (#Hs00426835_g1), *ADIPOQ* (#Hs00977214_m1), *BCL2* (#Hs00608023_m1), *BCL6* (#Hs00153368_m1), *GAPDH* (#Hs02758991_g1), *TAGLN* (#Hs01038777_g1), *CTFG* (#Hs00170014_m1), *CSF3* (#Hs99999083_m1), *CXCL10* (#Hs00171042_m1), *IL1β* (#Hs01555410_m1), *HAS1* (#Hs00758053_m1) *NANOG* (#Hs04260366_g1), *LEPTIN* (#Hs00174877_m1), *PTCH1* (#Hs00181117_m1), *KLF4* (#Hs00358836_m1), *OPN* (#Hs00959010_m1), *RUNX2* (#Hs01047973_m1), *PPARγ* (#Hs01115513_m1), *MYC* (#Hs00153408_m1), *IL6* (#Hs00174131_m1), *IL8* (#Hs00174103_m1), *CXCL1* (#Hs00236937_m1), *CXCL2* (#Hs00601975_m1), *CXCL3* (#Hs00171061_m1), *CCL2* (#Hs00234140_m1), *VEGFC* (#Hs01099203_m1), *FGF1* (#Hs01092738_m1), FGF2 (#Hs00266645_m1), *LIF* (#Hs01055668_m1), *ZEB1* (#Hs01566408_m1), TWIST1 (#Hs04989912_s1), *SNAI1* (#Hs00195591_m1), *SNAI2* (#Hs00161904_m1), *SOX2* (#Hs04234836_s1), *SOX9* (#Hs00165814_m1), *EPCAM* (#Hs00901885_m1), *STAT3* (#Hs00374280_m1), *POU5F1* (#Hs00999632_g1).

For transcriptomic analysis, the total RNAs were extracted from ln/ob-dT/aT bASCs from five different patients per condition or from BT474/MDA-MB-231 cells directly co-cultured for up to 14 days. The co-cultured breast cancer cells were sorted by FACS with classical MCS surface markers CD90 and CD73, and an epithelial surface marker CD24. The extraction was performed by using EXTRACTME Total RNA Kit, according to manual instructions (7Bioscience GmbH, Hartheim, Germany) and without DNase digestion. A total amount of 1 μg RNA per sample was sent to Novogene Co., Ltd. (Beijing, China) and used as input material for the RNA sample preparations. Sequencing libraries were generated using the NEBNext^®^ Ultra^TM^ RNA Library Prep Kit for Illumina^®^ (NEB, USA), following the manufacturer‘s recommendations. Differential expression analysis between two conditions/groups (five biological replicates per condition) was performed using the DESeq2 R–package and 60,450 genes were analyzed. The genes found by DESeq2 were further depicted in different pathways by GO enrichment analysis and KEGG pathway enrichment analysis. Heatmaps, volcano plots, violin plots and pathway analyses were either generated by Novogene Co., Ltd. (Beijing, China) or by re-analyzing the data using Microsoft^®^Excel^®^ (Redmond, Washington, USA) and GraphPad Prism 9 (GraphPad software Inc., USA).

**1.6 Human cytokine array and ELISA**

For cytokine measurement, bASCs in passage 5 were cultured for 3 days to a confluence of 90%, starved for 72 h, and the supernatants were harvested. The levels of chemokines, cytokines and growth factors in the conditioned media were determined by using a Human Cytokine array (RayBiotech Life, Peachtree Corners, GA, USA, #AAH-CYT-1000-8-RB) according to the manufacturer’s instructions. The signal intensities of chemiluminescent membranes were assessed by using a scoring system. The signal intensities of the positive controls were defined as 10, and the intensities of the negative controls were designated as 0, and illustrated as two color heatmap.

For ELISA analysis, cells were seeded into 6-cm dishes (360,000 cells per dish), starved for up to 72 h and the supernatant was collected. The following ELISA assays were used: CXCL10 (PeproTech, Rocky Hill, NJ, USA, #900-M39), CSF3 (PeproTech, Rocky Hill, NJ, USA, #900-M77), IL1β (PeproTech, Rocky Hill, NJ, USA, #900-K95), VEGF-C (DRG Diagnostic, Marburg, Germany, #EIA-4825), FGF/FGF2 (R&D Systems, Minneapolis, MN, USA, #DFB50), CCL2 (R&D Systems, Minneapolis, MN, USA, #DCP00), CXCL1-3 (RayBiotech Life, Peachtree Corners, GA, USA, #ELH-GRO-1), LIF (RayBiotech Life, Peachtree Corners, GA, USA, #ELH-LIF-1), IL6 (Proteintech^®^, Planegg-Martinsried, Germany, #KE00007), IL8 (Proteintech^®^, Planegg-Martinsried, Germany, #KE00006). All ELISA assays were performed as instructed.

**1.7 Spheroid formation, mitotic index, spheroid proliferation and EMT**

MCF-7, MDA-MB-231, MDA-MB-361 and BT474 cell spheroids were formed as described [1], with modifications. Cells with 75% confluency were harvested, counted, resuspended in 20 µL (100 to 500 cells/µL: MDA-MB-231, 5^10x3^; MCF-7, 7.5^10x3^; BT474, 7.5^10x3^ cells) of complete medium, and seeded onto the bottom of a lid of a 60 mm culture dish as hanging droplets. The lid was then put back onto the PBS-filled bottom chamber of the culture dish and incubated for 72 to 96 h (BT474 and MDA-MB-361: 72 h; MDA-MB-231 and MCF-7: 96 h) until spheroids were stably formed. Single spheroids were carefully picked with a 100 µL tip and transferred on Nunc Lab-Tek II CC2 chamber slides (Thermo Fisher Scientific, Waltham, USA). The spheroids were stained for phospho-histone H3 (pHH3, Ser10), α-tubulin and evaluated microscopically after 6 to 12 h of reattachment. To analyze the effect on breast cancer spheroids, bASCs were seeded as a bASCs feeder layer (ln/ob-dT/aT bASCs) for up to 96 h. Breast cancer cells/bASCs hybrid spheroids were formed with 15%, 30% or 50% of bASCs for a concentration kinetic. The ratio of 85% of breast cancer cells and 15% of bASCs was used for generating spheroids for all functional assays. The spheroid area, diameter, mitotic index and cell count were analyzed using ImageJ 1.49i software (National Institutes of Health, USA).

The proliferation of hybrid spheroids or breast cancer spheroids (MCF-7 and MDA-MB-231) cultivated with 15% bASC supernatant was analyzed by flow cytometry. Both cell lines were stained with CellTrace™ violet cell proliferation kit (Thermo Fisher Scientific, Waltham, USA, #C45557). The cells were seeded with an equal cell number, after 7 days, cells were analyzed using a FACSCalibur^TM^ (BD Bioscience, Heidelberg, Germany). The cell number of the control condition served as reference point and the proliferation was presented as fold change.

EMT was induced in BT474/MCF7 cell lines by indirect co-culture with bASCs (ln/ob-dT/aT) in a transwell system for up to 14 days. bASCs were seeded on the upper 6-well culture insert (35.000 cells/well) and breast cancer cells (25.000 cells/well) were seeded on the bottom of the 6-well. The medium contained 50% bASC medium and 50% BT474 or MCF7 medium. The cells were stained with antibodies against E-cadherin and N-cadherin, and DAPI for immunofluorescence quantification. The immunofluorescence intensities of both cell-cell junction proteins and the cell size were analyzed using ImageJ 1.49i software (National Institutes of Health, USA). The fluorescence background was measured in each individual image and was subtracted from the measured values.

**1.8 Motility assay**

For motility assay, cells were seeded in 24-well plates with low confluency and imaged at 5-min intervals for 13 h. After the cells completely attached to the 24-well plates, 15% of bASC supernatant was added into the wells. All time-lapse imaging was performed with an AxioObserver.Z1 microscope (Zeiss), imaged with an AxioCam MRc camera (Zeiss) equipped with an environmental chamber to maintain proper environmental conditions (37°C, 5% CO_2_). The time-lapse movies were analyzed by using ImageJ 1.49i software (National Institutes of Health, USA) with the manual tracking plugin, and Chemotaxis and Migration Tool (Ibidi GmbH, Germany). Tracks were derived from raw data points and were plotted in GraphPad Prism 9 (GraphPad software Inc., USA). The accumulated distance was calculated by using the raw data points by the Chemotaxis and Migration Tool. Thirty random cells per experiment were analyzed and the experiments were repeated independently three times.

The patterns of motility were evaluated as descripted [2]. The migration velocity was calculated as the mean sum of all accumulated distances divided by the migration time (equation. 1). The unit is given in micrometers per minutes.

Equation 1: $Migration velocity=\frac{1}{n}\sum_{i=1}^{n} \frac{di,accum}{t}$

The directionality is measured by comparing the Euclidian distance (di, euclid) to the accumulated distance (di, accum). Therefore, it represents a calculation of the directness of the cell trajectories (equation. 2). The directness is a parameter to characterize the straightness of migration. The value of the directionality is given as a ratio between the Euclidian distance and the accumulated distance simplified as [d/D].

Equation 2: $Directionality=\frac{1}{n}\sum_{i=1}^{n} \frac{di,euclid}{di,accum}$

**1.9 Spheroid cell viability, foci analyses and live/dead cell assay**

Control spheroids containing only breast cancer cells BT474, MCF7 or MDA-MB-231 or hybrid spheroids containing bASCs and breast cancer cells were formed. Subsequently, cell viability was measured via CellTiter-Blue^®^ assay (Promega GmbH, Walldorf, Germany, #G808B) as instructed, with low attachment 96-well plates to maintain the spheroid structure. Spheroids were treated with tamoxifen (TMX) (5 µM) or docetaxel (DTX) (50 nM) for up to 96 h.

The quantification of nuclear foci was performed with BT474 control or BT474/bASCs hybrid spheroids. Spheroids were formed, transferred into low attachment 24-well plates, and treated with DTX (50 nM) for 72 h. Floating spheroids were stained for γ-H2AX and 53BP1 for microscopic quantification. The following parameters were analyzed: double positive cells, cells with 20 ≥ foci, and number of double positive foci per cell.

The live/dead cell assay was conducted by using BT474 control or BT474/bASCs hybrid spheroids. The spheroids were cultured on a low attachment 96-well plate, treated with DTX (50 nM) for 72 h, and stained with the LIVE/DEAD viability/cytotoxicity kit (Thermo Fisher Scientific, Waltham, USA, #L3224) for 30 min and inspected using a fluorescence microscope. The polyanoionic dye calcein (490/515 nm) is retained on extracellular amines, whereas the propidium iodide dye (535/617 nm) stains the intracellular nucleic acids in dead cells. The spheroid surface area as well as the fluorescence intensity ratio were calculated by using ImageJ 1.52i software (National Institutes of Health, USA).

**2. References**

1. Ritter A, Roth S, Kreis NN, Friemel A, Hoock SC, Souto AS, Eichbaum C, Neuhoff A, Chen Q, Solbach C *et al*: **Primary Cilia in Trophoblastic Cells Potential Involvement in Preeclampsia**. *Hypertension* 2020, **76**(5):1491-1505.

2. Ritter A, Friemel A, Kreis NN, Louwen F, Yuan J: **Impact of Polo-like kinase 1 inhibitors on human adipose tissue-derived mesenchymal stem cells**. *Oncotarget* 2016, **7**(51):84271-84285.

**3. Supplemental tables and figures**

**Supplementary table 1:** Clinical information of breast cancer patients, whose mammary adipose tissues were used for bASC isolation.

|  | Pat. no. | BC type | Grade | Size | Nodes | Metas-  tasis. | Receptors | Ki67 | Pre-treated | Other diseases |
| --- | --- | --- | --- | --- | --- | --- | --- | --- | --- | --- |
| Lean | 13 | 7 NST,  5 inv. lob.,  1 inv. duct. | 2 G1  9 G2  2 G3 | 8 T1  4 T2  1 T4b | 10 N0  3 N1 | 4 MX  9 M0 | 7 ER^+^,  4 ER^+^PR^+^,  2 trip. neg. | 1 5%  7 15-20%  4 30%  1 90% | None | 1 T1 DM  2 A. HTN  1 MS  1 hypo-thyroidism  1 T2 DM |
| Obese | 11 | 8 NST,  3 inv. lob. | 4 G1  7 G2 | 9 T1  2 T2 | 11 N0 | 5 MX  6 M0 | 4 ER^+^,  7 ER^+^PR^+^ | 2 5%  6 10-15%  2 20-25%  1 35-45% | 1 TMX  1 RTx | 2 A. HTN  2 HTN  1 T2 DM  1 polyarthritis |

Abbreviation: Pat. no: patient numbers; KI67: proliferation marker Ki-67, NST: no special type; inv. lob.: invasive lobular; inv. duct.: invasive ductal; ER: estrogen receptor; PR: progesterone receptor; trip. neg.: triple negative; TMX: tamoxifen; RTx: radiotherapy; T1/T2 D: type 1/2 diabetes mellitus; A. HTN.: arterial hypertension; MS: multiple sclerosis.

| **Supplementary table 2:** Cell surface markers of different lean bASCs | | | | | | |
| --- | --- | --- | --- | --- | --- | --- |
|  | | | | | | |
| **bASC No.:** | **CD90** | **CD73** | **CD146** | **CD14** | **CD31** | **CD106** |
| ln-aT I | 92.47 | 97.64 | 37.54 | 0.22 | 0.48 | 1.4 |
| ln-dT I | 77.11 | 96.73 | 46.79 | 0.1 | 8.42 | 0.5 |
| ln-aT II | 86.33 | 99.92 | 11.88 | 0 | 6.02 | 1.1 |
| ln-dT II | 90.09 | 99.97 | 24.8 | 2.128 | 0.1 | 11.82 |
| ln-aT III | 87.54 | 87.74 | 12.66 | 0.2 | 6.86 | 0.03 |
| ln-dT III | 73.86 | 80.97 | 0.05 | 0.02 | 12.84 | 9.64 |
| ln-aT IV | 80.6 | 91.87 | 0.12 | 0.38 | 0.15 | 8.2 |
| ln-dT IV | 98.95 | 85.74 | 0.18 | 0.02 | 9.48 | 5.96 |
| ln-aT V | 95.58 | 98.54 | 38.15 | 0.68 | 0.94 | 0.58 |
| ln-dT V | 89.66 | 94.01 | 84.59 | 3.22 | 0.64 | 0.54 |
| ln-aT VI | 96.55 | 94.38 | 44.31 | 0.44 | 18.7 | 5.13 |
| ln-dT VI | 97.67 | 91.37 | 75.24 | 2.6 | 2.02 | 1.86 |
| **ln-aT MV:** | **89.85** | **95.02** | **24.11** | **0.32** | **5.53** | **2.74** |
| **ln-dT MV:** | **87.89** | **91.47** | **38.61** | **1.35** | **5.58** | **5.05** |
| Abbreviation: CD, cluster of differentiation; MV, mean value. | | | | | | |

| **Supplementary table 3:** Cell surface markers of different obese bASCs | | | | | | |
| --- | --- | --- | --- | --- | --- | --- |
|  | | | | | | |
| **bASC No.:** | **CD90** | **CD73** | **CD146** | **CD14** | **CD31** | **CD106** |
| ob-aT I | 63.83 | 95.24 | 37.35 | 0.00 | 4.30 | 3.22 |
| ob-dT I | 92.06 | 96.31 | 15,14 | 0.00 | 5.11 | 1.31 |
| ob-aT II | 91.02 | 99.67 | 93.70 | 1.43 | 2.17 | 2.60 |
| ob-dT II | 87.14 | 99.55 | 98.22 | 7.62 | 0.73 | 3.90 |
| ob-aT III | 69.40 | 84.42 | 0.43 | 0.01 | 12.82 | 0.50 |
| ob-dT III | 91.08 | 88.37 | 1.56 | 0.02 | 2.94 | 0.04 |
| ob-aT IV | 71.44 | 98.78 | 33.83 | 2.45 | 0.16 | 0.10 |
| ob-dT IV | 98.64 | 96.27 | 95.44 | 0.58 | 3.11 | 1.01 |
| ob-aT V | 99.06 | 99.31 | 94.66 | 0.81 | 0.21 | 0.44 |
| ob-dT V | 96.39 | 92.04 | 18.33 | 2.57 | 2.50 | 2.14 |
| ob-aT VI | 97.67 | 91.37 | 75.24 | 1.47 | 2.60 | 5.73 |
| ob-dT VI | 87.44 | 91.58 | 93.28 | 1.94 | 2.54 | 2.09 |
| **ob-aT MV:** | **82.07** | **94.80** | **55.87** | **1.03** | **3.71** | **2.10** |
| **ob-dT MV:** | **92.13** | **94.02** | **61.37** | **2.12** | **2.82** | **1.75** |
| Abbreviation: CD, cluster of differentiation. | | | | | | |

**Supplementary table 4:** Clinical information of breast cancer patients, whose mammary adipose tissues were used for IHC staining.

|  | Pat. no. | BC type | Grade | Cancer size | Nodes | Meta-stasis | Receptors | Ki67 | Pre-treated | Age at diagnosis |
| --- | --- | --- | --- | --- | --- | --- | --- | --- | --- | --- |
| Lean  (BMI: 22.56± 2.56) | 16 | 1 NOS,  2 inv. lob.,  13 inv. duct. | 12 G2  4 G3 | 13 T3p  3 T4p | 2 N0  7 N1  3 N2  4 N3 | 15 M0  1 M1 | 8 ER^+^,  4 ER^+^PR^+^,  4 trip. neg. | 1 10%  4 15%  2 30%  9 n.d. | 6 neo-adjuvant therapy | 63.88 ± 11.70 |
| Obese  (BMI: 33.80± 6.12) | 16 | 1 IBC,  1 inv. lob.,  14 inv. duct. | 9 G2  7 G3 | 10 T3p  6 T4p | 3 N0  6 N1  3 N2  4 N3 | 15 M0  1 M1 | 11 ER^+^,  3 ER^+^PR^+^  2 trip. neg. | 1 10%  2 15%  1 25%  1 40%  1 80%  10 n.d. | 8 neo-adjuvant therapy | 52.06 ± 13.45 |

Abbreviations: Pat. no: patient numbers; NOS: not otherwise specified; IBC: inflammatory breast cancer; Ki67: proliferation marker Ki-67; inv. lob.: invasive lobular; inv. duct.: invasive ductal; ER: estrogen receptor; PR: progesterone receptor; trip. neg.: triple negative; n.d.: not determined.

**
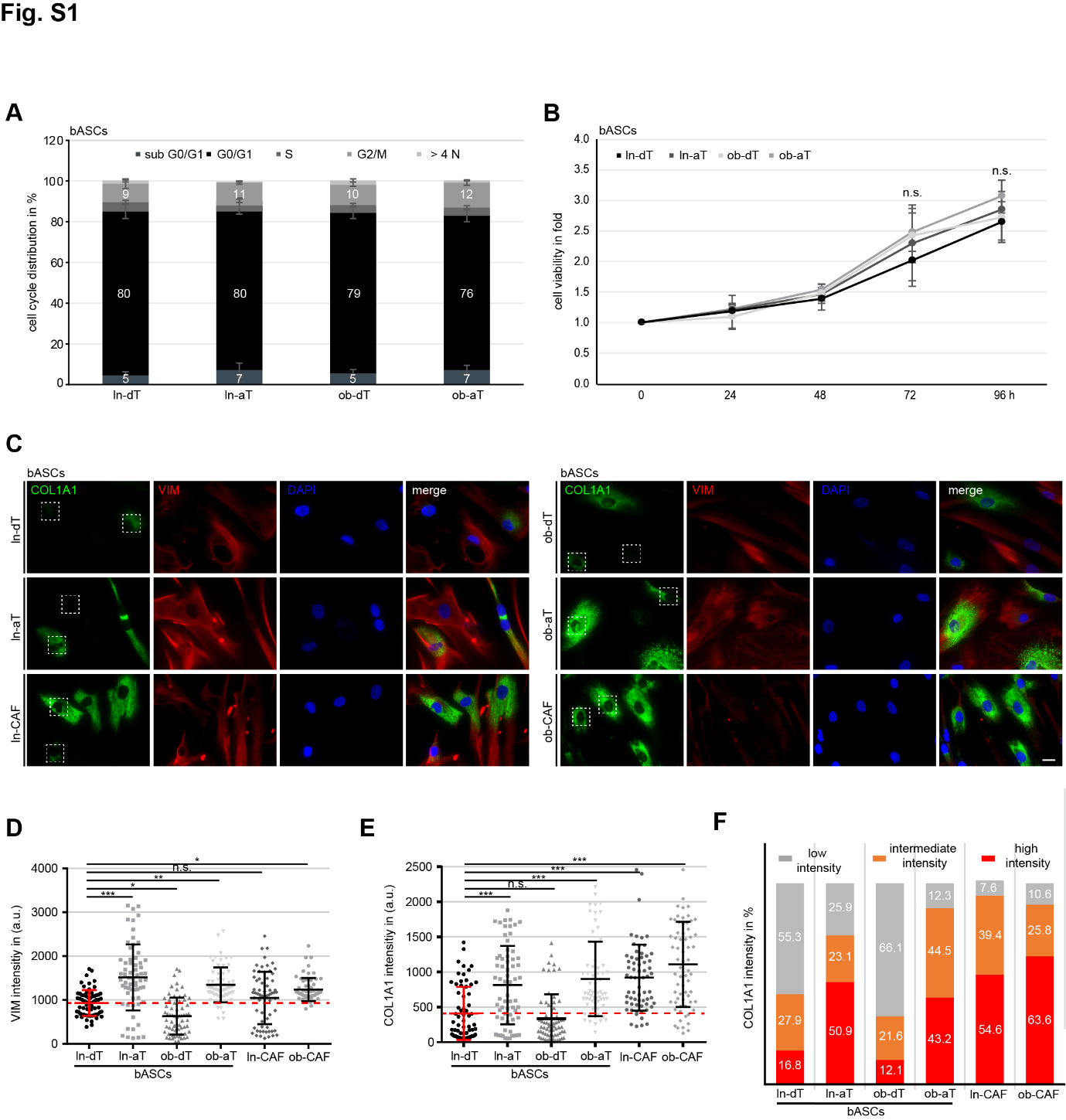
**

**Figure S1: No obvious alterations in cell cycle distribution and cell viability among various bASC subgroups, and more COLA1 and vimentin in aT-bASCs than in dT-bASCs.**

(**A**) Cell cycle distribution was analyzed using a FACSCalibur^TM^. The cell cycle phases of different bASC subgroups (ln-dT, ln-aT, ob-dT and ob-aT) are presented in percentage and the results were derived from three independent experiments. (**B**) bASCs were seeded in 96-well plates for 0, 24, 48, 72 and 96 h. Cell viability was measured via CellTiter-Blue^®^ assay. The results are from three independent experiments and presented as mean ± SEM. Student’s t-test was used. n.s., p > 0.05. (**C**) bASCs (ln-dT, ln-aT, ob-dT and ob-aT) were stained for COL1A1, vimentin (VIM), and DNA for further quantification. Representative images of bASCs and CAFs (ln-/ob CAFs) are shown. Scale: 20 μm. (**D-F**) The evaluation of the mean fluorescence intensity of VIM (D) and COL1A1 (E) in bASCs and CAFs are presented as scatter plots. The results are from three independent experiments (n = 3, 90 cells for each condition and in each group) and presented as mean ± SEM. Unpaired Mann-Whitney U test was used in (D and E). ∗p < 0.05, ∗∗p < 0.01, ∗∗∗p < 0.001. (F) Quantification of the COL1A1 staining signal in three intensity subgroups (low < 350, intermediate 350-800, high > 800 a.u.) (n = 3, 90 cells for each condition and in each group).


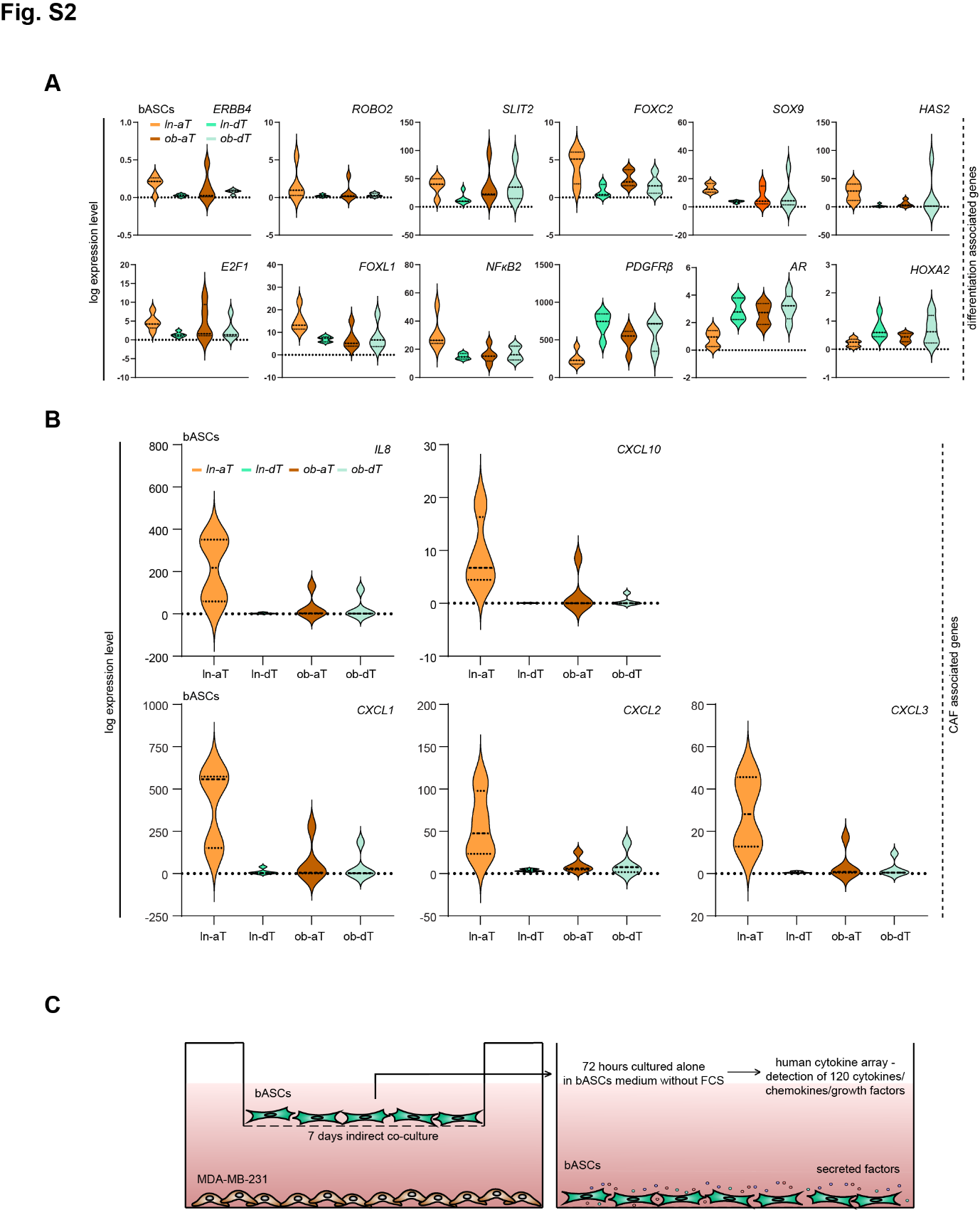


**Figure S2: Both ln- and ob-aT bASCs display deregulated differentiated associated genes and ln-aT bASCs increased iCAF genes.**

(**A and B)** Total RNAs were extracted from bASC subgroups (ln-dT, ln-aT, ob-dT and ob-aT, each 5 samples) for whole genome transcriptome analysis. Violin plots present selected differentially expressed genes associated with cancer cell stemness (A) or iCAF marker genes (B). Values reflect the log expression levels (FPKM, Fragments Per Kilobase Million) of genes from the RNA-seq data. (**C**) Experimental setup: bASCs were seeded in the upper chamber of a transwell system in bASC medium, MDA-MB-231 cells were seeded as monolayer at the bottom of a transwell system with appropriate medium for 7 days. Afterwards, bASCs were transferred in a new chamber for additional 72 h in FCS free bASC medium. The supernatant was used for a human cytokine array.


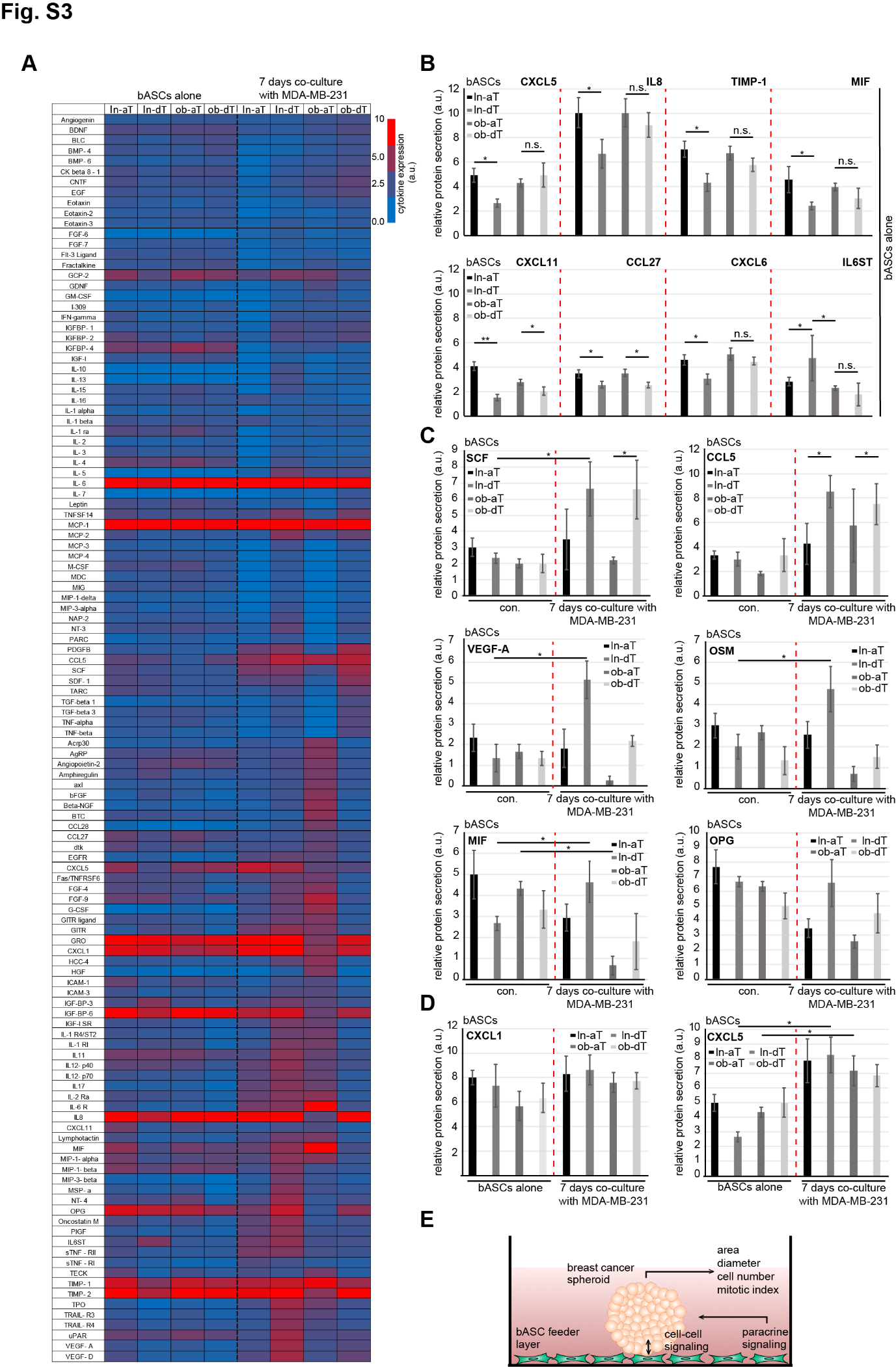


**Figure S3: Secretion of bASCs alone or upon incubation with MDA-MB-231 cells.**

(**A-D)** Conditioned media were taken from bASCs alone or bASCs indirectly incubated with MDA-MB-231 cells for 7 days for measuring the secretion of cytokine/chemokine via a human cytokine array. (A) The heatmap presents the collective analysis of 120 cytokines/chemokines in the supernatants of the indicated bASC subgroups. Human cytokine array plots were used for a semi-quantitative analysis. Blue color represents a score of 0 and red color indicates a score of 10. (B**)** Bar graphs show relative quantification of cytokines (CXCL5, Il8, TIMP-1, MIF, CXCL11, CCL27, CXCL6 and IL6ST) secreted by bASCs cultured alone. Student’s t test was used. ∗p < 0.05. (C and D) Bar graphs show relative quantifications of indicated cytokines (SCF, CCL5, VEGF-A, OSM, CXCL1 and CXCL5) secreted by bASCs cultured alone or indirectly co-cultured with MDA-MB-231 for 7 days. The results (A-D) are from three independent experiments (n = 3, three individually collected conditioned media were used for each group). Student’s t test was used. ∗p < 0.05. (**E**) Experimental setup. bASCs were seeded as monolayer on plastic tissue culture dishes (2D) and breast cancer spheroids (3D) were cultured on top of the bASC feeder layer with control medium for breast cancer cells.


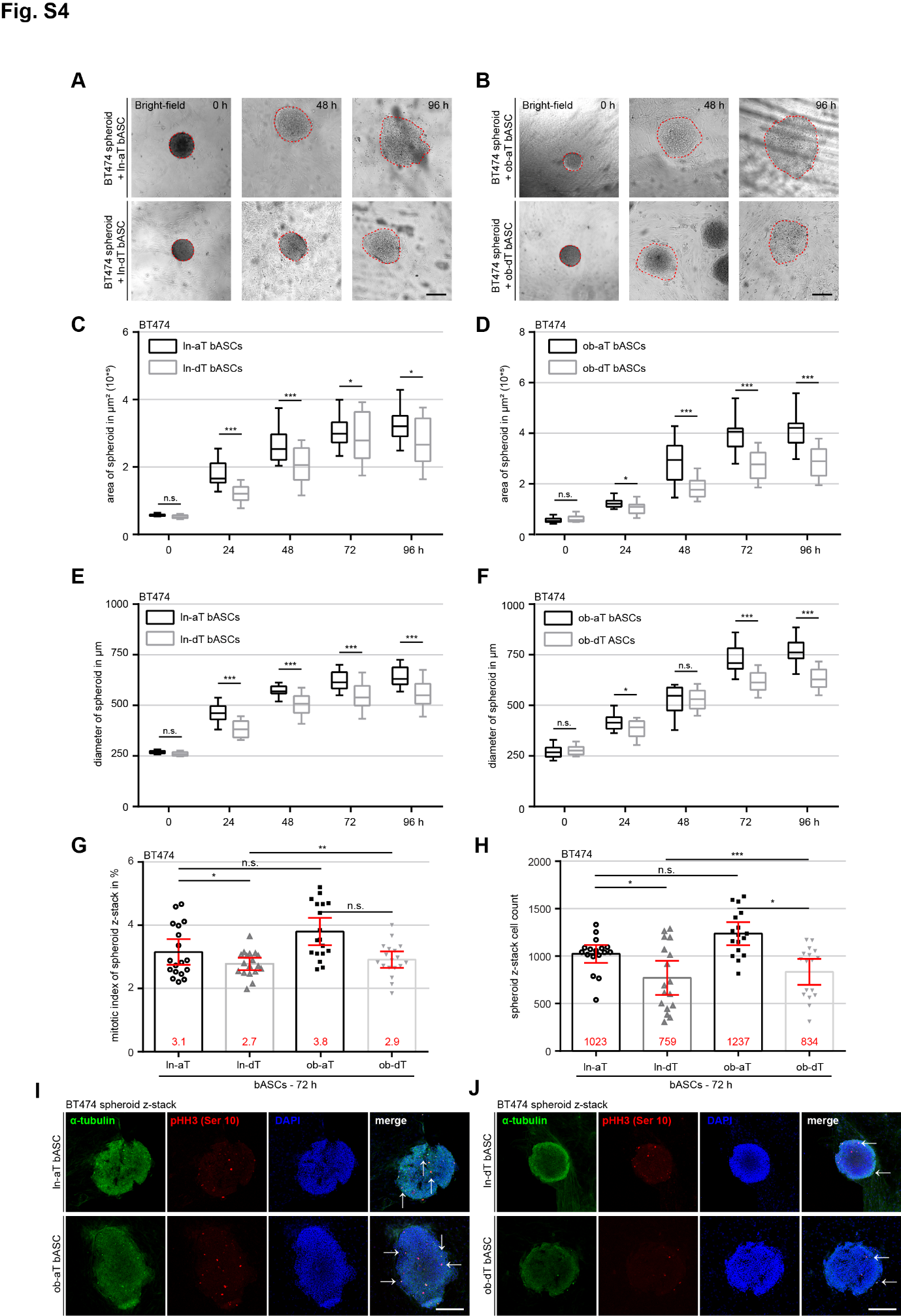


**Figure S4: ob- and ln-aT bASCs promote the growth of BT474 spheroids.**

BT474 spheroids were generated as hanging drops for 24 h. Spheroids were directly co-cultured with different bASCs (ln-dT, ln-aT, ob-dT and ob-aT) for up to 96 h and analyzed microscopically. (**A-B**) Representative bright-field images showing BT474 spheroids co-cultured with indicated bASCs for 0 h, 48 h and 96 h. Scale: 250 μm. Red dotted lines depict the measured area. (**C-F**) The area and diameter of BT474 spheroids were evaluated after co-culture with ln-aT or ln-dT bASCs (C and E) and ob-aT or ob-dT bASCs (D and F). The results are based on three individual experiments (n = 30 spheroids for each group) and presented as box plots showing median ± min/max whiskers. (**G-J**) BT474 spheroids were co-cultured for 72 h, stained for α-tubulin (green), pHH3 (Ser10) (red) and DNA (DAPI, blue) for further analysis. (G and H) Quantification of the mitotic index (G) and spheroid cell number (H) are shown as bar scatter graphs. The results are from three independent experiments (n = 18 spheroids for each condition) and presented as mean ± SEM. (I and J) Representative images of BT474 spheroids co-cultured with indicated bASC subgroups are shown. Scale bar: 20 μm. Unpaired Mann-Whitney U test was used. ∗p < 0.05, ∗∗p < 0.01, ∗∗∗p < 0.001.

**
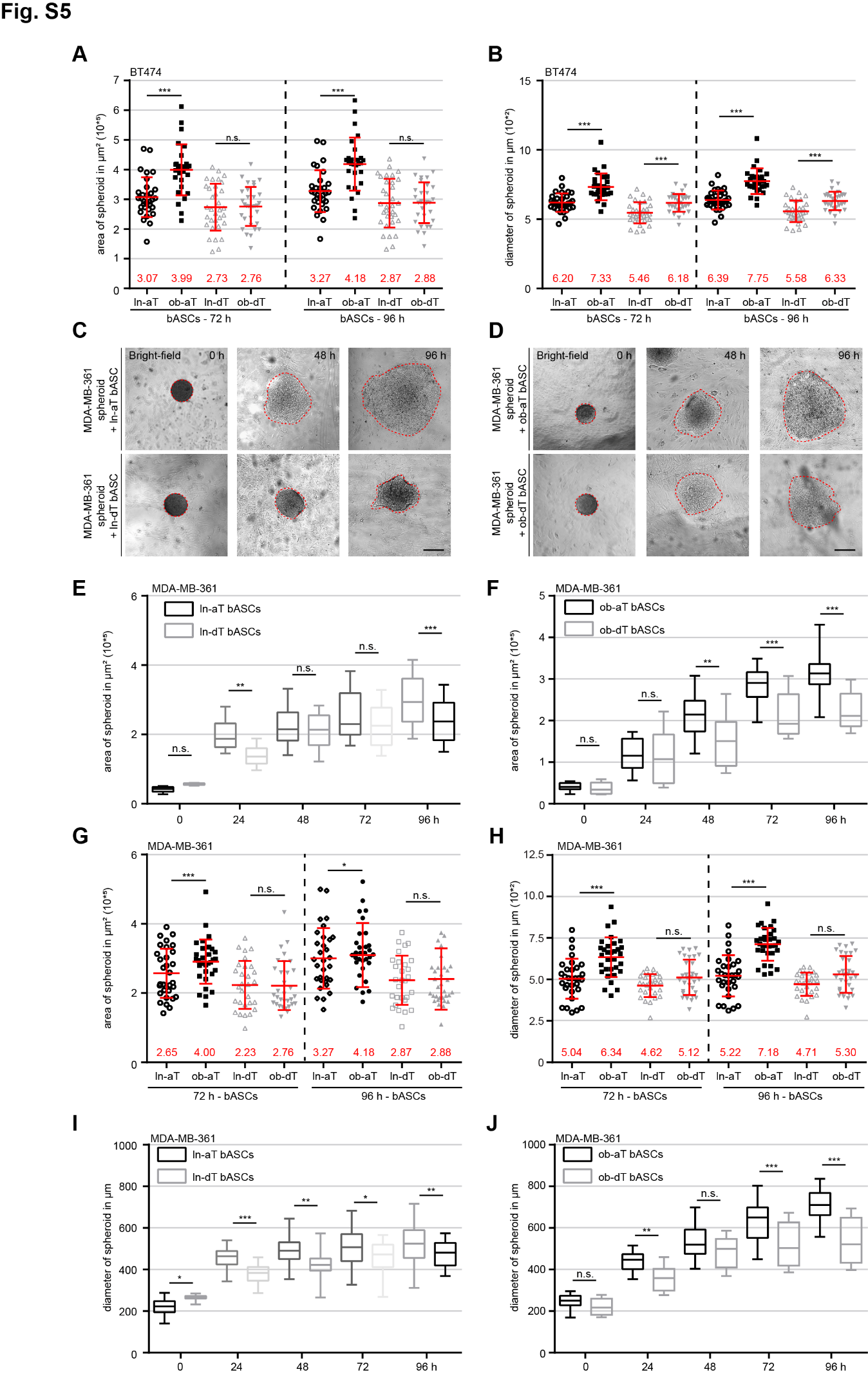
Figure S5: Direct co-culture of ob- and ln-aT bASCs promotes the growth of BT474 and MDA-MB-361 spheroids.**

BT474 or MDA-MB-361 spheroids were generated as hanging drops for 24 h. Spheroids were directly co-cultured with different bASCs (ln-dT, ln-aT, ob-dT and ob-aT) for up to 96 h and analyzed microscopically. **(A and B)** The area and diameter of co-cultured BT474 spheroids were evaluated. The results are based on three individual experiments (n = 30 spheroids for each group) and presented as box plots showing median ± min/max whiskers. **(C and D)** Representative bright-field images showing MDA-MB-361 spheroids directly co-cultured with indicated bASCs for 0, 48 and 96 h. Scale: 250 μm. Red dotted lines depict the measured area. **(E-J)** The area and diameter of MDA-MB-361 spheroids were evaluated after direct co-culture with ln-aT or ln-dT bASCs. The results are based on three individual experiments (n = 30 spheroids for each group) and presented as box plots showing median ± min/max whiskers. Unpaired Mann-Whitney U test was used. ∗p < 0.05, ∗∗p < 0.01, ∗∗∗p < 0.001.


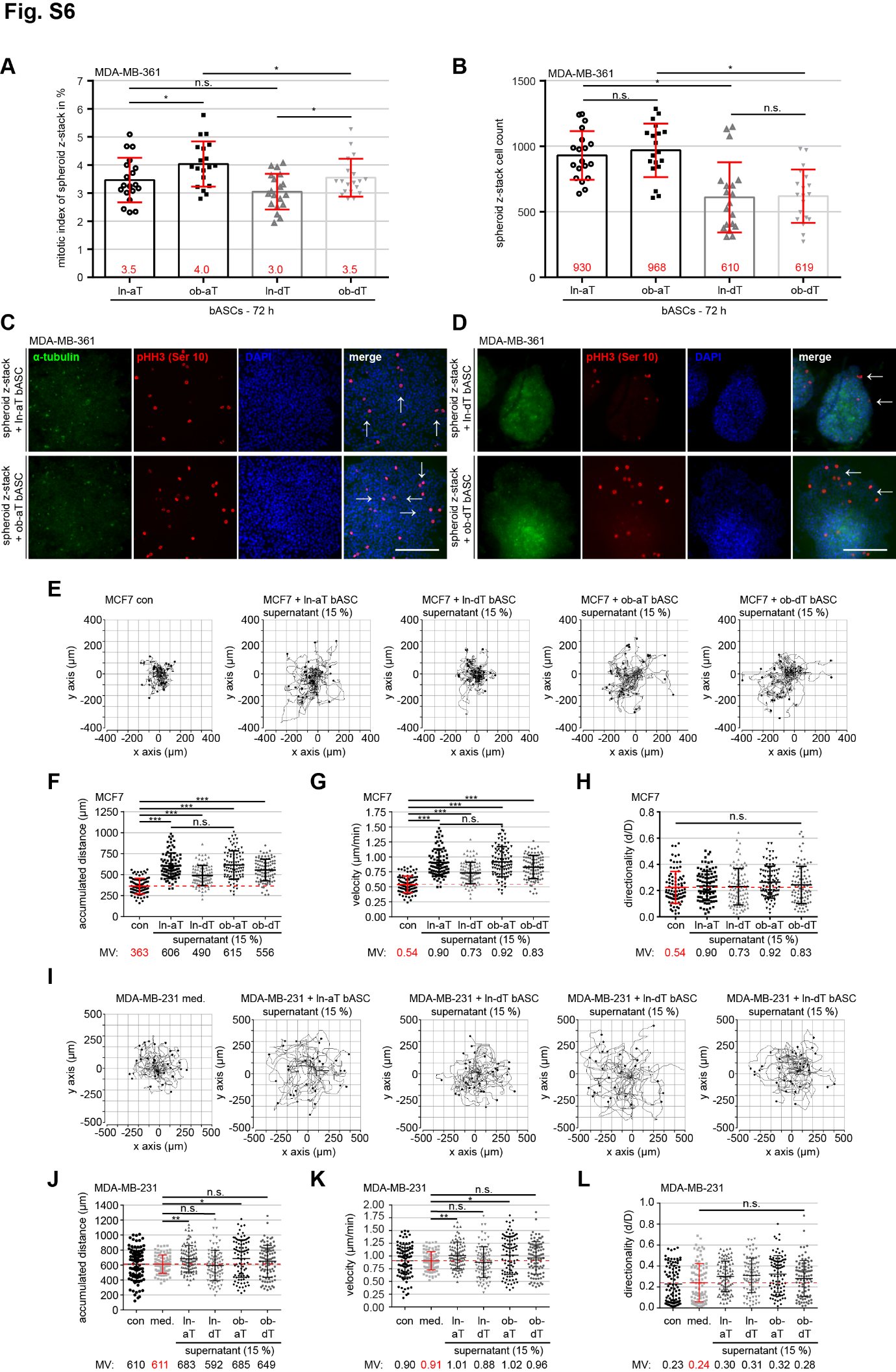


**Figure S6: Ob- and ln-aT bASCs promote proliferation of MDA-MB-361 spheroids and their supernatants enhance the motility of breast cancer cells.**

(**A-D**) MDA-MB-361 spheroids were directly co-cultured for 72 h and stained for α-tubulin (green), pHH3 (Ser10) (red) and DNA (DAPI, blue) for quantification. (A and B) Quantification of the mitotic index (A) and spheroid cell number (B) are shown as bar scatter graphs. The results are from three independent experiments (n = 18 spheroids for each condition) and presented as mean ± SEM. (C and D) Representative images of MDA-MB-361 spheroids directly co-cultured with indicated bASCs subgroups are shown. Scale bar: 20 μm. Unpaired Mann-Whitney U test was used. ∗p < 0.05, ∗∗p < 0.01, ∗∗∗p < 0.001. (**E-L**) Single MCF7 (E-H) or MDA-MB-231 (I-L) cultured with 15% supernatant of different bASCs (ln-dT, ln-aT, ob-dT and ob-aT) were tracked using time-lapse microscopy to analyze their motility. (E and I) Representative trajectories are depicted for individual MCF7 (E) or MDA-MB-231 cells (I) (n = 30 cells in each group). The accumulated distance (F and J), velocity (G and K) and directionality (H and L) were evaluated and the results from three experiments are shown as scatter plot with mean ± SEM (n = 90 cells). Unpaired Mann-Whitney U test was used. *p < 0.05, **p < 0.01, ***p < 0.001.


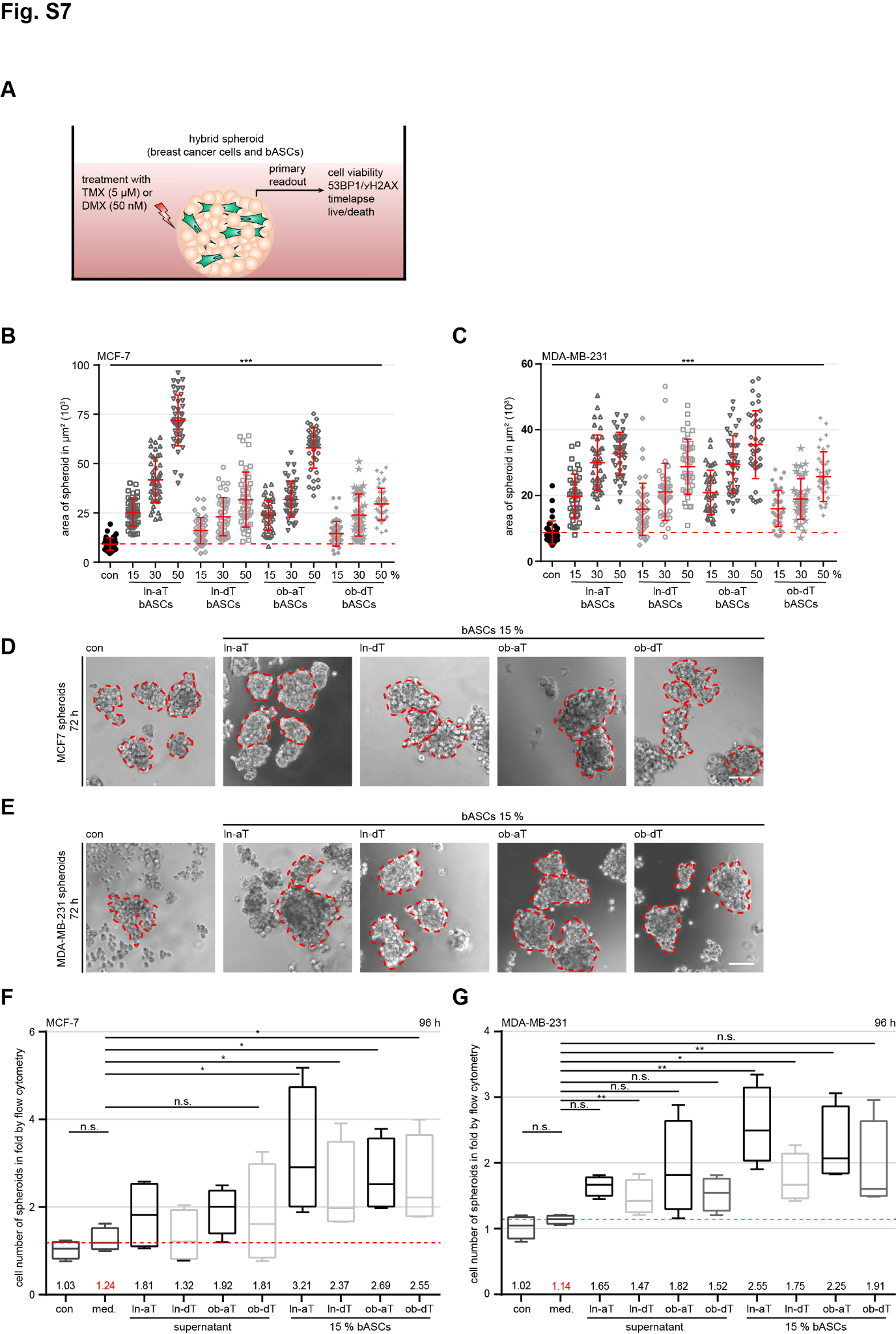


**Figure S7: bASCs promote the formation and growth of spheroids of breast cancer cells.**

(**A**) Experimental setup. Breast cancer cell/bASC hybrid spheroids (3D) were generated in individual ratios for indicated experiments. (**B-G**) Spheroids from MCF7 (B, D and F) and MDA-MB-231 cells (C, E and G) alone or integrated with bASCs as hybrid spheroids were generated for 72 h. Their area was microscopically evaluated (B and C) and cell count in fold change was analyzed by FACS (F and G). The quantification of the area is based on three individual experiments (n = 90 spheroids for each group) and presented as scatter plots showing mean value ± SEM (B and C). Representative bright-field images showing MCF7 hybrid spheroids (D) or MDA-MB-231 hybrid spheroids (E) directly co-cultured with indicated bASCs for 72 h. Scale: 100 μm. Red dotted lines depict the measured area. The cell count of individual spheroid conditions was measured by staining *CellTrace™* violet after 96 h, which was quantified by FACS. The results are based on three individual experiments (F and G). Unpaired Mann-Whitney U test was used in (B and C). ∗∗∗p < 0.001. Student’s t test was used in F and G. ∗p < 0.05, ∗∗p < 0.01.


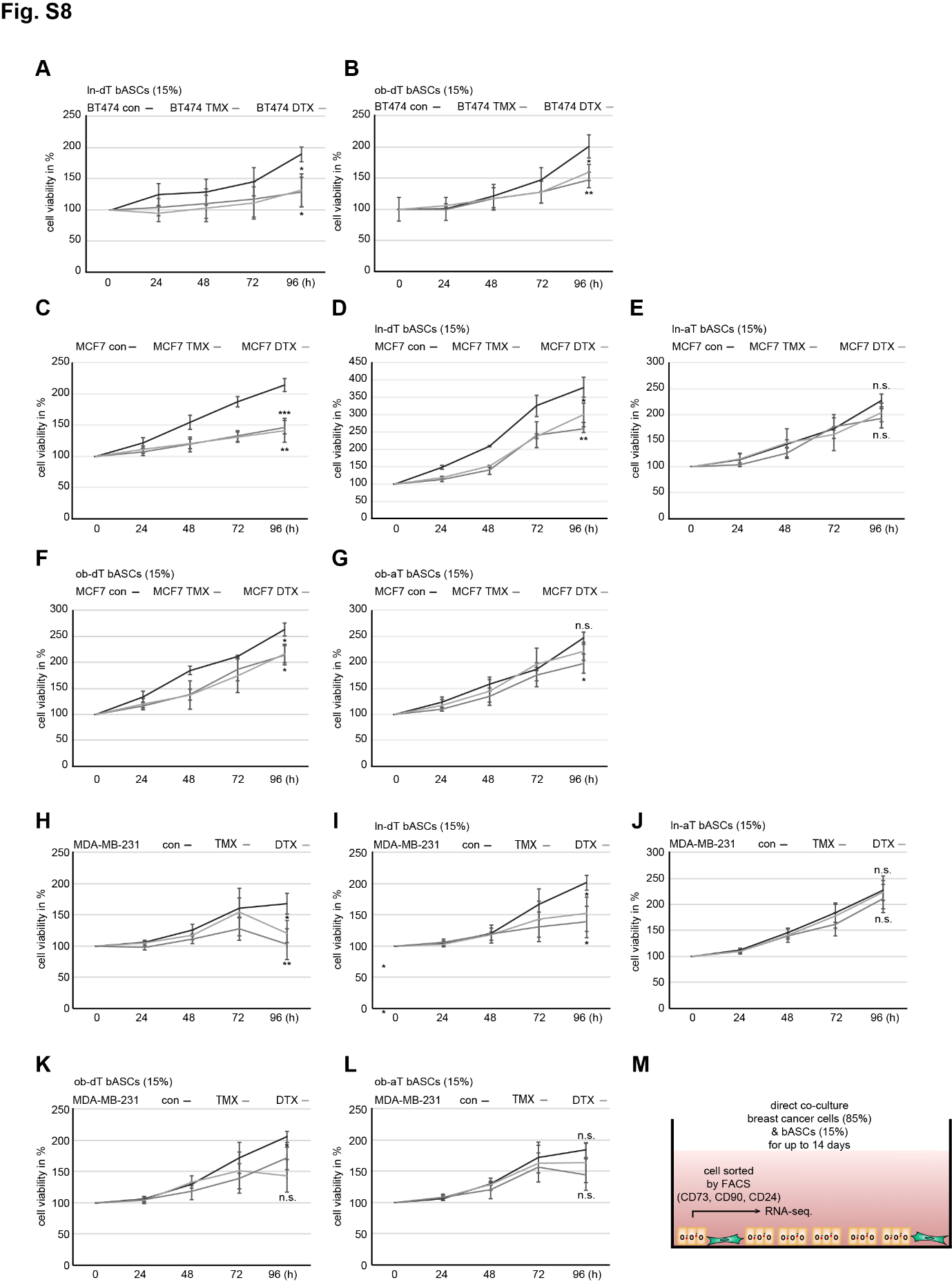


**Figure S8: Hybrid spheroids of breast cancer cells and bASCs demonstrate an increased resistance to chemotherapeutic agents.**

(**A-L**) BT474 (A and B), MCF7 (C-G) and MDA-MB-231(H-L) spheroids or hybrid spheroids integrated with 15% of ln-aT, ln-dT, ob-aT or ob-dT bASCs were generated for 24 h, transferred into 96-well low-attachment plates, and treated with DMSO, TMX (5 µM) or DTX (50 nM) for up to 96 h. Cell viability was measured at indicated time points. The results are based on three independent experiments and presented as mean ± SEM. Student’s t test was used. ∗p < 0.05, ∗∗p < 0.01, ∗∗∗p < 0.001. (**M**) Experimental setup: bASCs and breast cancer cells were seeded as mixed monolayer (2D) with direct cell-cell contact for up to 14 days. Cells were then sorted using the cell surface markers CD90/CD73/CD24 for further experiments.


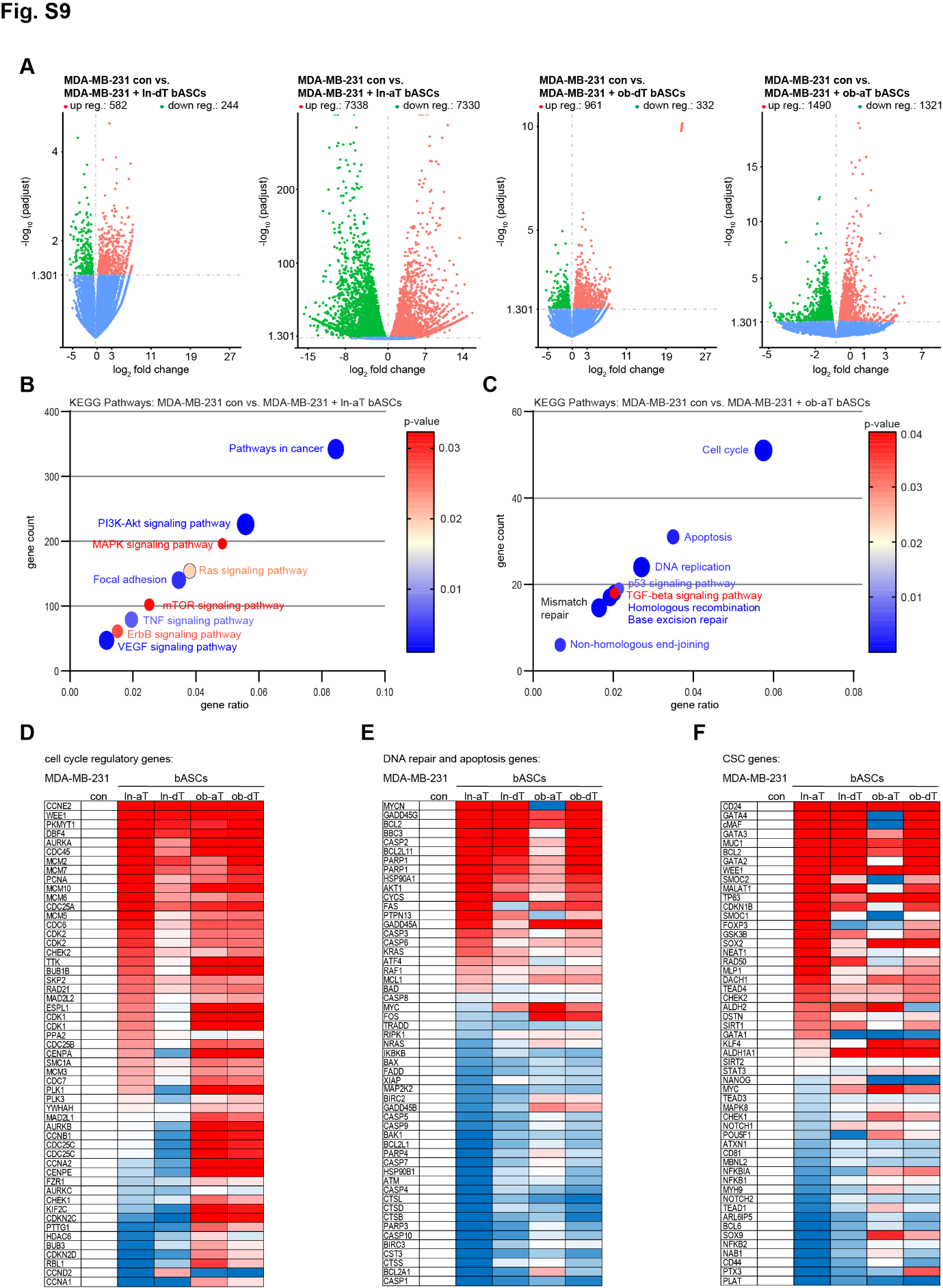


**Figure S9: Transcriptome profiles of triple negative MDA-MB-231 cells directly co-cultured with bASCs.**

MDA-MB-231 cells cultured alone or in direct co-culture with different bASCs (ln/ob-dT/aT) for 14 days were sorted for RNA-seq analysis (n = 3). The gene expression was analyzed using DESeq2 R package. **(A)** Volcano plots show the adjusted p-values for genes differentially expressed between control MDA-MB-231 cells and MDA-MB-231 cells directly co-cultured with ln-dT bASCs (1^st^ volcano plot), ln-aT bASCs (2^nd^ volcano plot), ob-dT bASCs (3^rd^ volcano plot) and ob-aT bASCs (4^th^ volcano plot). **(B and C)** KEGG pathway enrichment in RNA-seq with a p-value < 0.05. Gene count: the number of genes that are deregulated in the pathway. Gene ratio: ratio of the number of target genes divided by the number of all genes in each pathway. Significance is color coded (high p-value, red; low p-value, blue). **(D-F)** Heatmaps present deregulated genes involved in cell cycle regulation (D), DNA repair and apoptosis (E) and CSC (F). A fold change of ≥ 2 (red color code) and ≤ -2 (blue color code) are shown. Included genes have a p-adjusted value of < 0.05 for at least one condition (ln-aT or ob-aT).


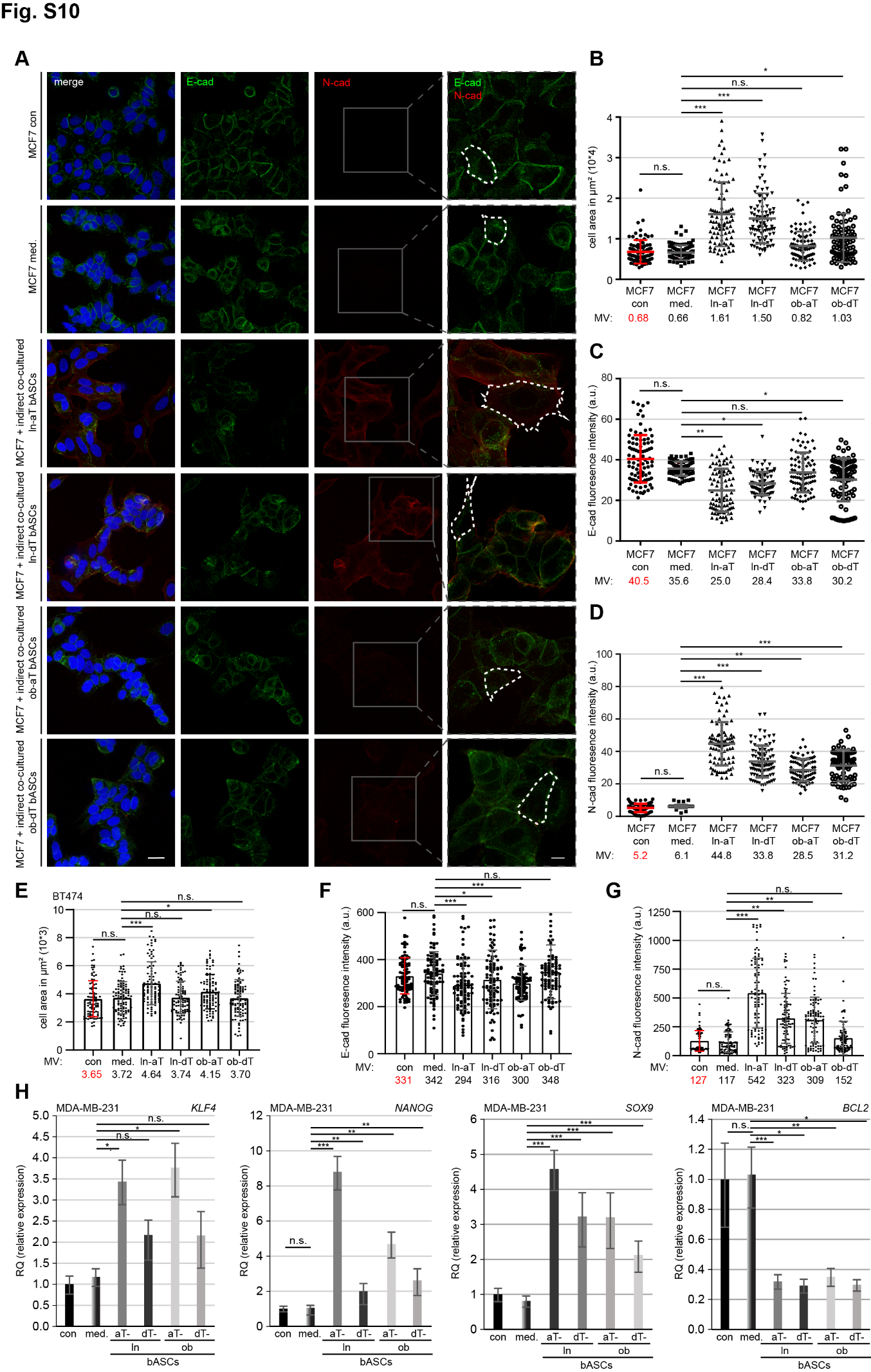


**Figure S10: ln-aT bASCs efficiently trigger EMT in epithelial breast cancer cell lines, while ob-aT bASCs more efficiently stimulate MDA-MB-231 cells into a CSC phenotype.**

(**A-G**) MCF7 (A-D) or BT474 (E-G) cells cultured alone or in indirect co-culture with different bASCs (ln/ob-dT/aT) for 14 days were stained for E-cadherin (E-cad, green, epithelial marker), N-cadherin (N-cad, red, mesenchymal marker) and DNA (DAPI, blue). (A) Representative images of MCF7 cells are shown, with indicated culture conditions. Scale: 25 μm. Inset scale: 10 μm. White dotted lines depict the measured areas. (B-G) The area (B and E), E-cadherin fluorescence intensity signal (C-F) and N-cadherin fluorescence intensity signal (D-G) of MCF7 or BT474 cells were evaluated after co-culture. The results are based on three individual experiments (n = 90 cells for each group) and presented as scatter plots (B-D) showing median ± min/max whiskers or scatter bar plots showing mean and SEM (E-G). Unpaired Mann-Whitney U test was used. ∗p < 0.05, ∗∗p < 0.01, ∗∗∗p < 0.001. (**H**) MDA-MB-231 cells directly co-cultured with different bASCs (ln/ob-dT/aT) for 14 days were sorted for qPCR analysis. Relative gene levels of CSC-associated genes *KLF4*, *NANOG*, *SOX9* and *BCL2* are shown for MDA-MB-231 cells co-cultured with different bASC subgroups (ln-dT, ln-aT, ob-dT and ob-aT). The results are from three independent experiments and presented as bar graphs with mean ± SEM. Student’s t test was used. *p < 0.05, **p < 0.01, ***p < 0.001.
